# TFAP2A is a component of the ZEB1/2 network that regulates TGFB1-induced epithelial to mesenchymal transition

**DOI:** 10.1186/s13062-017-0180-7

**Published:** 2017-04-17

**Authors:** Yoana Dimitrova, Andreas J. Gruber, Nitish Mittal, Souvik Ghosh, Beatrice Dimitriades, Daniel Mathow, William Aaron Grandy, Gerhard Christofori, Mihaela Zavolan

**Affiliations:** 1 Biozentrum, University of Basel, Klingelbergstrasse 50-70, CH-4056 Basel, Switzerland; 2 Department of Biomedicine, University of Basel, Mattenstrasse 28, CH-4058 Basel, Switzerland; 3 Department of Cellular and Molecular Pathology, German Cancer Research Center (DKFZ), Heidelberg, Germany

**Keywords:** Epithelial-to-mesenchymal transition, EMT, Transcription regulatory network, TFAP2A, ZEB2, TGFb1, NMuMG

## Abstract

**Background:**

The transition between epithelial and mesenchymal phenotypes (EMT) occurs in a variety of contexts. It is critical for mammalian development and it is also involved in tumor initiation and progression. Master transcription factor (TF) regulators of this process are conserved between mouse and human.

**Methods:**

From a computational analysis of a variety of high-throughput sequencing data sets we initially inferred that TFAP2A is connected to the core EMT network in both species. We then analysed publicly available human breast cancer data for TFAP2A expression and also studied the expression (by mRNA sequencing), activity (by monitoring the expression of its predicted targets), and binding (by electrophoretic mobility shift assay and chromatin immunoprecipitation) of this factor in a mouse mammary gland EMT model system (NMuMG) cell line.

**Results:**

We found that upon induction of EMT, the activity of TFAP2A, reflected in the expression level of its predicted targets, is up-regulated in a variety of systems, both murine and human, while TFAP2A’s expression is increased in more “stem-like” cancers. We provide strong evidence for the direct interaction between the TFAP2A TF and the ZEB2 promoter and we demonstrate that this interaction affects ZEB2 expression. Overexpression of TFAP2A from an exogenous construct perturbs EMT, however, in a manner similar to the downregulation of endogenous TFAP2A that takes place during EMT.

**Conclusions:**

Our study reveals that TFAP2A is a conserved component of the core network that regulates EMT, acting as a repressor of many genes, including ZEB2.

**Reviewers:**

This article has been reviewed by Dr. Martijn Huynen and Dr. Nicola Aceto.

**Electronic supplementary material:**

The online version of this article (doi:10.1186/s13062-017-0180-7) contains supplementary material, which is available to authorized users.

## Background

The epithelial to mesenchymal transition (EMT) is defined as the process in which cells that display predominantly epithelial features transition to a state in which they exhibit mesenchymal characteristics. EMT has well-established and important roles in different stages of embryonic development: it is observed during gastrulation, in the generation of the primitive mesoderm, during neural crest (NC) formation, and in the development of many organs such as heart valves, skeletal muscle, and the palate [1]. EMT-like phenomena were also described in adult organisms, as part of normal developmental changes, as well as during pathological processes [2]. For example, during breast development, an EMT-like program referred to as epithelial plasticity is thought to be part of branching morphogenesis, which leads to the formation of the complex ductal tree [3]. Recent findings suggest that an EMT program may increase the “stemness” potential of epithelial cells [4].

The mammary gland epithelium is composed of an internal luminal layer, and an external, basal layer of myoepithelial cells. Recent studies suggest that these different cell types derive from a common stem cell, through a process that involves epithelial plasticity [5, 6]. Whereas this process is very well coordinated in normal development, its dysregulation in cancer leads to outcomes that are difficult to predict [3]. While the majority of experimental results indicate that manipulating EMT also affects cancer metastasis, recent reports on cancer cells circulating in the blood stream or resulting from genetic lineage tracing have questioned a critical role of EMT in the formation of metastases, but have demonstrated a role in chemotherapy resistance [7–9]. In breast cancer, it is believed that EMT affects the basal epithelial phenotype and is responsible for an increased metastatic potential [10].

The TFAP2A transcription factor (TF) is expressed early in embryogenesis, where it contributes to cell fate determination in the formation of the neural crest and the epidermis. The knockout of Tfap2a in mouse is lethal due to neural crest formation defects [11]. In humans, mutations in *TFAP2A* have been linked to the developmental defects in the Branchio-Oculo-Facial Syndrome (BOFS) [12].

TFAP2A is a member of the AP-2 family of TFs, which in humans and mice is composed of five members, TFAP2A, TFAP2B, TFAP2C, TFAP2D and TFAP2E, or AP-2α, AP-2β, AP-2γ, AP-2δ and AP-2ε, respectively. These proteins share important sequence similarities and have a specific structural organization with a proline and glutamine-rich trans-activation domain located at the N-terminus, a central region with positively-charged amino acids, and a highly conserved helix-loop-helix region at the C-terminus. The last two domains are involved in DNA binding and dimerization, the proteins being able to form hetero- or homo-dimers [13]. The *TFAP2A* gene is composed of seven exons. In mice, four different isoforms have been described [14]. Systemic Evolution of Ligand by EXponential enrichment (SELEX)-based, in vitro assays, have determined that AP-2α binds to the palindromic motif GCCN_3_GGC and to some close variants, GCCN_4_GGC, GCCN_3/4_GGG [15]. More recent ChIP-seq experiments inferred SCCTSRGGS and SCCYSRGGS (S = G or C, R = A or G and Y = C or T) as the consensus sites for human AP-2γ and AP-2α, respectively [16].

In the adult mammary gland, TFAP2A is expressed in virgin and pregnant mice. Its mRNA and protein are detected at the terminal end buds and also in the ductal epithelium, predominantly in the luminal cell population [17]. Targeted overexpression of TFAP2A and TFAP2C in the mouse mammary gland results in lactation deficiency, increased proliferation and apoptosis, reduced alveolar budding and differentiation [17, 18]. Knockout of the TFAP2C paralog of TFAP2A in mouse mammary luminal cells results in an increased number of terminal end buds with reduced distal migration [19].

Aberrant expression of TFAP2A has been observed in various cancers. It is overexpressed in human nasopharyngeal carcinoma and is involved in tumorigenesis by targeting the HIF-1α/VEGF/PEDF pathway [20]. In contrast, reduced AP-2α expression was reported to be associated with poor prognosis in gastric adenocarcinoma [21]. The loss of TFAP2A is connected with the acquisition of the malignant phenotype in melanoma through regulation of cell adhesion molecules (ALCAM) [22]. TFAP2A expression was found to be less organized in breast cancer compared to normal mammary gland and it is associated with HER2/ErbB-2 and ERα expression [23].

To define conserved EMT regulatory networks, we started by analyzing seven mouse and human datasets obtained from EMT systems, altogether containing thirty-six mRNA sequencing samples. We found that TFAP2A is one of the factors that contribute most significantly to mRNA-level expression changes that take place during embryonic stem cell (ESC) differentiation to mesoderm or to NC cells, during normal mammary gland development, and most importantly, in breast cancer models. To investigate TFAP2A’s involvement in EMT we used mouse mammary gland epithelial cell line NMuMG, a well-known model of EMT [24]. We demonstrate, for the first time, that the expression and activity of *Tfap2a* are modulated during TGFβ1-induced transdifferentiation of these cells. We further show that TFAP2A directly binds to the *Zeb2* promoter, modulating its transcriptional output. TFAP2A overexpression in NMuMG cells results in increased levels of EMT-inducing TFs, and promotes an EMT-like phenotype. Our study sheds a new light on the role of TFAP2A in processes that involve EMT, including breast cancer, and it contributes to a deeper understanding of the molecular and cellular mechanism of cancer development and metastasis.

## Methods

### Expression vectors and constructs

Mouse TFAP2A cDNA was kindly provided by Prof. Qingjie [25]. The TFAP2A-FLAG fusion was subcloned into pDONR201 plasmid, using a Gateway® BP Clonase® II Enzyme mix (#11789-020, Life Technologies) and it was further subcloned into pCLX vector, using Gateway® LR Clonase® II Enzyme mix (#11791-020, Life Technologies).

### Cell culture

We used a subclone of NMuMG cells that was generated as previously described (NMuMG/E9) [24]. Cells were cultured in Dulbecco's modified Eagle's medium (DMEM #D5671, Sigma Aldrich) with high glucose and L-glutamine, supplemented with 10% fetal bovine serum (#f-7524, Sigma-Aldrich) and where indicated were treated with 2 ng/ml TGFβ1 (#240-B, R&D Systems). Transient transfection was done using Lipofectamine2000 (#11668-019, Life Technologies) according to the manufacturer's instructions. For time course experiments, cells were grown in six well plates for up to 14 days and treated with 2 ng/mL TGFβ1. In addition, NMuMG pCLX-TFAP2A or NMuMG pCLX-GFP cells induced with 2 μg/mL of doxycycline for 6 days, and further treated or not treated with TGFβ1 for 72 hours were used to study the effect of TFAP2A overexpression.

### Lentiviral infection

Stable populations of NMuMG cells expressing the blasticidine-resistant marker together with TFAP2A-FLAG under a doxycycline-inducible promoter were obtained with the pCLX expression system [26]. Lentiviral particles were produced in HEK293-LV cells using the helper vectors pMDL, pREV and the envelope-encoding vector pVSV. For infection, viral supernatants were added to target cells in the presence of polybrene (#TR-1003-G, Millipore) (1 μg/ml). Cells were further incubated at 37 °C under 5% CO_2_ in a tissue culture incubator for 72 h, prior to selection with blasticidine at 10 μg/ml (#15205-25 mg, Sigma-Aldrich).

### Light microscopy and immunofluorescence

Cells were treated with doxycycline or TGFβ1 for the indicated times, and were grown on gelatin coated glass coverslips. Cells were fixed with 4% paraformaldehyde in 1x PBS for 15 min (Fig. 2a, b). They were later permeabilized and blocked for 30 min with 0.1% Triton X-100 (#T8787, Sigma-Aldrich), 10% goat serum (#16210072, Gibco®, Life Technologies), and 1% BSA (#A9647, Sigma-Aldrich) in PBS (#20012-019, Gibco®, Life Technologies). Afterwards, the coverslips were incubated with the indicated primary antibodies overnight at 4 °C, and then with Alexa Fluor 488,647 conjugated secondary antibodies, (Molecular Probes, Life Technologies), for one hour at room temperature. Where appropriate, Acti-stain™ 555‬ (#PHDH1‬, Cytoskeleton)‬ diluted 1:200 was added together with secondary antibody stain. The coverslips were mounted with VECTASHIELD™ DAPI Mounting Media (Vector Laboratories) on microscope slides and imaged with a confocal microscope (Zeiss LSM 700 Inverted).

### Quantitative real-time reverse transcription PCR

Total RNA was extracted with TRI Reagent® (#T9424, Sigma-Aldrich) and further purified with Direct-zol™ RNA MiniPrep kit (#R2050, Zymo Research). Reverse transcription was performed with SuperScript® III Reverse Transcriptase (#18080-044, Life Technologies) according to the manufacturer’s instructions. For qPCR, 8 ng of cDNA was used in a reaction with Power SYBR® Green PCR Master Mix (#4367659, Applied Biosystems). Gene expression changes are normalized to the expression of the house-keeping genes *Gapdh* and *Rplp0*.

### mRNA sequencing

For the mRNA-seq library preparation, a well of a 6-well plate of NMuMG cells was used, either treated with growth factor and/or doxycycline, or with control reagents for the indicated times. mRNA-seq libraries were prepared as already described [27].

### Chromatin immunoprecipitation (ChIP), sequencing library preparation and data analysis

The ChIP protocol was adapted from [28]. Cells were crosslinked in fixing buffer (50 mM HEPES pH 7.5, 1 mM EDTA pH 8.0, 0.5 mM EGTA pH 8.0, 100 mM NaCl, 1% formaldehyde) for 10 min with continuous rocking at room temperature (RT), and then quenched with 125 mM glycine for 5 min. Cells were washed three times with cold PBS and collected by scrapping. Nuclei were isolated, and lysed to obtain crosslinked chromatin. Simultaneously, the antibody was coupled with protein G magnetic beads (#88848, Pierce™) by incubating 100 μl of protein G beads with 10 μg of TFAP2A-specific antibody (Novus) and 10 μg of rabbit IgG (#PP64, Millipore) as a negative control, for minimum 1 h at RT with continuous rotation. A probe sonicator was then used in cold conditions to reduce heating, for six cycles of 30 s pulse-on at amplitude value of 60 and 1 min and 15 s pulse-off to obtain chromatin fragments of 100–500 bp followed by centrifugation at 20,000 g for 10 min at 4 °C to get rid of nuclear debris. Further, 3% chromatin was kept as input control from each sample and an equal amount (around 750–1000 μg) of chromatin was incubated with magnetic beads-coupled antibody at 4 °C overnight with continuous rotation. Immuno-complexes were washed with 1 mL of wash buffers as described in the original protocol. Samples of washed immuno-complexes along with the input were further treated with RNase and then with proteinase K followed by overnight reverse crosslinking at 65 °C with continuous shaking at 1400 rpm in a thermoblock with heating lid. DNA was purified using Agencourt AMPure XP (#A63880, Beckman Coulter) beads as detailed in the reference. The enrichment of specific target genes was quantified by qRT-PCR, comparing the TFAP2A-ChIP with the IgG negative control.

Libraries of ChIPed and input DNA were prepared according to the instruction manual of NEBNext® ChIP-Seq Library Prep Reagent Set for Illumina. In brief, end repair of input and ChIPed DNA was done by incubating with T4 DNA Polymerase Klenow fragment and T4 PNK enzyme at 20 °C for 30 min. The reaction was purified using Ampure beads according to the instruction manual. An A nucleotide overhang at the 3’ end was produced by treating the end repaired DNA with dATP and Klenow Fragment (3´ → 5´ exo^−^) at 37 °C for 20 min followed by DNA purification. Double stranded DNA adapters were ligated to dA overhang DNA by T4 DNA ligase reaction at 37 °C for 30 min followed by DNA purification and size selection as described in the instruction manual. Size selected DNA was PCR-amplified for 16 cycles using NEBNext® High-Fidelity 2X PCR Master Mix with Illumina universal forward primer and indexed reverse primer, that enabled multiplexing of samples for sequencing. Amplified DNA was finally purified and sequenced on an Illumina Hiseq2500 instrument. The obtained sequencing reads were mapped to the genome and visualized within the clipz genome browser (www.clipz.unibas.ch).

### Antibodies and reagents

We used primary antibodies against the following proteins: TFAP2A (#sc-12726, Santa Cruz Biotechnology) for Western Blot (WB) and TFAP2A (#NBP1-95386, Novus Biologicals, Bio-Techne) for immunofluorescence and immunoprecipitation, actin (#sc-1615, Santa Cruz Biotechnology), E-cadherin (#610181, BD Transduction Laboratories), N-cadherin (#610921, BD Transduction Laboratories), Fibronectin (#F3648, Sigma-Aldrich), GAPDH (#sc-32233, Santa Cruz Biotechnology), vimentin (#v2258, Sigma-Aldrich). Recombinant human TGFβ1 was obtained from R&D Systems.

### Electrophoretic Mobility Shift Assay (EMSA)

TnT® T7 Quick Coupled Transcription/Translation System (#L1171, Promega) was used to express in vitro translated TFAP2A from the pcDNA3-TFAP2A construct. Double-stranded oligonucleotide probes were end-labeled with ^32^P and purified on autoseq G-50 columns (#27-5340-01, Amersham). Binding reactions containing probe, TFAP2A protein, poly (dI-dC) (#81349, Sigma-Aldrich) non-specific competitor in gel retention buffer (25 mM HEPES pH 7.9, 1 mM EDTA, 5 mM DTT, 150 mM NaCl, 10% Glycerol) and electrophoresis were carried out as described previously [29].

### Combined motif activity response analysis

The datasets used in the following analysis are listed in Additional file 1: Table S1. We applied the ISMARA tool to each dataset as previously described [30]. Briefly, the Motif Activity Response Analysis (MARA) infers the activity of regulatory motifs from the number of binding sites of each motif *m* in each promoter *p* (*N*_*m,p*_) and the genome-wide expression driven by these promoters *p* in samples *s* (*E*_*p,s*_):

$$ {E}_{p, s}={\tilde{c}}_s+{c}_p+{\displaystyle \sum_m}{N}_{m, p}{A}_{m, s} $$

where ${\overset{\sim }{c}}_s$ represents the mean expression in sample s, ${c}_p$ is the basal expression of promoter *p*, and ${A}_{m, s}$ is the (unknown) activity of motif *m* in sample *s*. To identify motifs that consistently change in activity across datasets we used a computational strategy as previously described [31]. In brief, first we obtained the average activities over the replicates of each condition in every dataset. Next, because the range of gene expression levels and consequently the motif activities varied across datasets, we re-centered and then standardized the averaged motif activities ${\overline{A}}_{m, g}^{*}$ and corresponding errors ${\overline{\sigma}}_{m, g}^{*}$, belonging to a specific condition *g*. To standardize the activities in a given dataset with the epithelial-like condition labeled as *a* and the mesenchymal-like condition by *b* we defined a scaling factor $\mathrm{S}=\sqrt{\frac{{\left({\overline{A}}_{m, g}^{* b}\right)}^2+{\left({\overline{A}}_{m, g}^{* a}\right)}^2}{2}}$, and then rescaled the activities ${\overset{\sim }{A}}_{m, g}^{*}=\frac{{\overline{A}}_{m, g}^{*}}{\mathrm{S}}$ and the corresponding errors ${\overset{\sim }{\sigma}}_{m, g}^{*}=\frac{{\overline{\sigma}}_{m, g}^{*}}{\mathrm{S}}$. Subsequently, we separated the condition-specific, averaged and rescaled activities (${\overset{\sim }{A}}_{m, g}^{\ast }$) and errors (${\overset{\sim }{\sigma}}_{m, g}^{\ast }$) obtained from different datasets into two groups, depending on whether they originated from epithelial-like cells (*a*) or mesenchymal-like cells (*b*). We averaged activities belonging to the same group as done for sample replicates before (see above and [31]). Finally, to rank motif activity changes during EMT we calculated for every motif *m* a z-score by dividing the change in averaged activities by the averaged errors:

$$ \mathrm{z}=\frac{{\overline{\mathrm{A}}}_{m, g}^{\ast b}-{\overline{\mathrm{A}}}_{m, g}^{\ast a}}{\sqrt{{\left({\overline{\sigma}}_{m, g}^{\ast b}\right)}^2+{\left({\overline{\sigma}}_{m, g}^{\ast a}\right)}^2}} $$

### Constructing motif-motif interaction networks

ISMARA predicts potential targets for each motif *m* by calculating a target score *R* as the logarithm of the ratio of two likelihoods: the likelihood of the data *D* assuming that a promoter *p* is a target of the motif, and the likelihood of the data assuming that it is not:

$$ R= log\left(\frac{P\left( D\Big| target\  promoter\right)}{P\left( D\Big| not\  target\  promoter\right)}\right) $$

The posterior probability *p* that a promoter is a target given the data and assuming a uniform prior of 0.5 is given by $p=\frac{1}{1+\frac{1}{e^R}}$. To construct motif-motif interactions, we focused on those transcription regulators, whose regulatory regions were consistently (within all datasets) predicted by ISMARA to be targeted by motifs of other regulators. We obtained a combined probability *p*_*comb*_ that a regulator is a target of a particular motif *m* across *I* different datasets by calculating the probability product of the probabilities obtained from individual datasets:

$$ {p}_{comb}={\displaystyle \prod_{i=1}^I}{p}_i $$

### GOBO analysis

The top 100 target genes of the TFAP2 {A,C}.p2 motif as derived by applying ISMARA to the Neve et al. data set [32] were analyzed with the Gene Expression-Based Outcome for Breast Cancer Online (GOBO) tool [33]. For each gene only the promoter with the highest ISMARA target score was considered for the analysis.

### Estimating gene expression log_2_ fold changes from mRNA sequencing data

For each sample *s* the expression values driven by each promoter of a gene *g* (determined by ISMARA, see above) were summed up to estimate the expression of gene *g* in sample *s*. Log_2_ gene expression fold changes were then calculated for TGFβ1-treated pCLX-GFP (pCLX-GFP + TGF-beta), pCLX-TFAP2A (pCLX-TFAP2A), and for TGFβ1-treated pCLX-TFAP2A (pCLX-TFAP2A + TGF-beta) cell lines relative to the pCLX-GFP (pCLX-GFP) control cells.

## Results

### TFAP2A/C motif activity increases upon EMT in both mouse and human systems

Aiming to identify major regulators of EMT and to further construct a conserved network of their interactions, we used the Motif Activity Response Analysis (MARA) approach, which combines high-throughput measurements of mRNA expression with computational prediction of regulatory elements [30]. The published ISMARA tool [30] allows not only the automated analysis of individual data sets, but also the inference of motifs that most generally explain gene expression changes across multiple experiments.

**Fig. 1:**
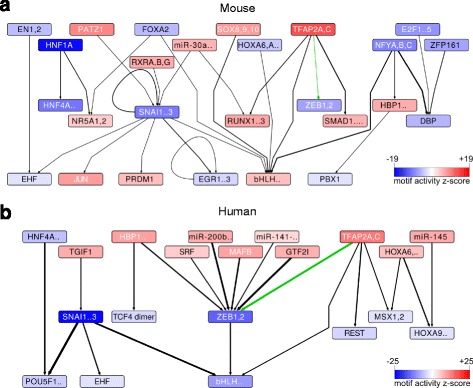
The transcriptional networks inferred from different EMT systems. Motif–motif interaction networks derived from mouse (**a**) and human (**b**) datasets. An *arrow* was drawn from a motif A to a motif B if motif A was consistently (across datasets from the corresponding species) predicted to regulate a transcriptional regulator *b* that is known to bind motif B. The probability product that A targets *b* is reflected by the thickness of the line. For readability, only motifs with an absolute z-score > 2.0 and having at least one interaction with another such motif (with a target probability product > 0.35 for human and > 0.15 for mouse) are depicted. The color intensity of the nodes representing motifs is proportional to the significance of the motif given by its z-score. *Red* indicates increased and *green* indicates decreased activity upon EMT

The results from the combined MARA analysis of different EMT mRNA expression datasets from breast epithelial cell lines of mouse and human, and from the differentiation of human pluripotent stem cells into NC cell and mesoderm (Additional file 1: Table S1) are shown in Fig. 1 [34–40]. How much a given motif contributes to the observed gene expression changes is quantified in terms of a combined z-score, which in our case represents the significance of the motif activity change between the epithelial and mesenchymal cell types (denoted by the intensity of the color in Fig. 1a and b and listed in Additional file 1: Tables S2 and S3). Based on the genome-wide computational prediction of binding sites for transcription regulators we can further infer motif interaction networks. In Fig. 1, an arrow is drawn between two motifs A and B when any of the regulators that recognizes motif B is a predicted target of motif A. The motif interaction networks derived from mouse and human EMT models suggest that only a small fraction of the TFs has a highly conserved and significant role in both species. The core transcriptional network of EMT, containing the TFs *Zeb1*, *Zeb2* and *Snai1*, is conserved, as expected. The motifs that correspond to these factors have negative activity changes during EMT (represented by the blue color on the scheme) which indicates that the expression of their targets decreases, as expected from their known repressive function during the process [41]. The TFAP2A/C motif is also a conserved component of both mouse and human EMT networks. Its target genes are upregulated during EMT (reflected by the red color in the figure) and thus the motif itself is predicted to have a highly significant positive change in activity. Furthermore, in both human and mouse systems, the TFAP2A/C motif is predicted to target both *Zeb1* and *Zeb2* TFs (Fig. 1a and b).

### TFAP2A expression and activity changes in EMT and breast cancer

**Fig. 2:**
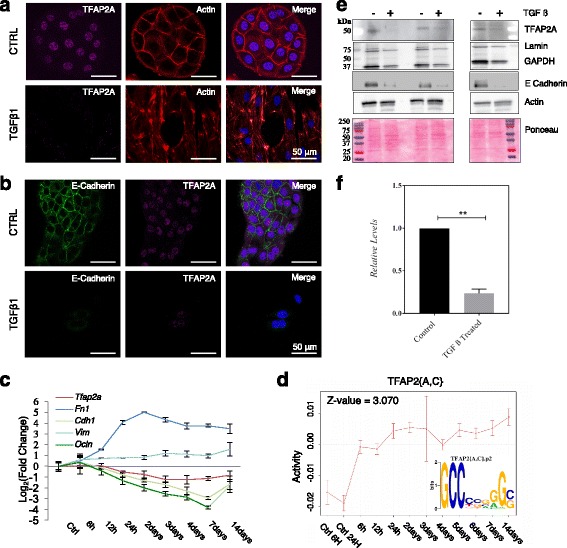
TFAP2A expression and activity profile in the NMuMG EMT model. **a**-**b** NMuMG cells were treated with 2 ng/mL of TGFβ1 for 72 h and were stained for TFAP2A and F-Actin (**a**) and TFAP2A and E-cadherin (**b**). The merged panels represent colocalization of the imaged markers with the nucleus which was stained with DAPI and compared to controls. Scale bar represents 50 μm. **c** NMuMG cells were treated for 14 days with 2 ng/mL of TGFβ1. Quantitative RT-PCR of Tfap2a during the time course of this treatment indicates that Tfap2a mRNA levels are reduced upon EMT. The EMT markers E-cadherin (Cdh1), Fibronectin (Fn1), Occludin (Ocln), and Vimentin (Vim) follow the expected trend. **d** Two mRNA-seq samples from independent wells were prepared from a time course of NMuMG cells treated for 14 days with 2 ng/mL of TGFβ1, and the data was consequently analyzed with ISMARA [30]. The figure depicts the dynamics of TFAP2A/C transcriptional activity during the time course. The sequence logo of the TFAP2A/C binding motif is also indicated. **e**-**f** Lysates from NMuMG/E9 cells treated with 2 ng/mL of TGFβ1 for 72 h were probed for TFAP2A, GAPDH and Lamin B expression by WB and their levels compared with the expression levels of Actin and also to the Ponceau-stained membrane (**e**). The bar plot represents the densitometric quantification of the TFAP2A protein levels upon treatment compared to the control (**f**) ** indicates a *p*-value < 0.01 in the paired *t*-test (*P* = 0.0014)

We made use of the murine mammary gland cell line NMuMG to further investigate the role of the AP-2 family members TFAP2A and TFAP2C in EMT. Upon induction with TGFβ1, NMuMG cells undergo EMT, which manifests itself through E-cadherin downregulation, formation of actin stress fibers and an elongated, mesenchymal-like cell shape (Fig. 2a, b and [36]). mRNA-seq revealed that of the five members of the AP-2 family, only *Tfap2a* is expressed in this system, with reads covering all its exons (Additional file 1: Figure S1). Immunofluorescence staining of endogenous TFAP2A demonstrated that the protein has a predominantly nuclear localization (Fig. 2a, b). 48 h after the TGFβ1 stimulation we observed that *Tfap2a* mRNA levels decreased moderately and further declined during the 14 days time course, while the common EMT markers such as E-cadherin, Fibronectin and Vimentin followed the expected trend (Fig. 2c).

We next generated mRNA-seq data from a 14 days time course of NMuMG cells stimulated with 2 ng/mL TGFβ1. Applying ISMARA to these data revealed the dynamics of TFAP2A activity during the entire length of the time course (Fig. 2d). As the paralogous TFAP2A and TFAP2C bind similar sequences, we therefore refer to their shared binding motif as TAFP2 {A,C}. In contrast to its mRNA expression (Fig. 2c), the TFAP2A transcriptional activity, reflected in the behavior of its targets, increases during EMT (Figs. 1 and 2d). This indicates that TFAP2A probably acts as a repressor in this context. Despite the fact that *Tfap2a* transcript levels and the TAFP2{A,C} motif activity exhibit a clear negative correlation, we observed the highest increase in activity in the first 6 h of treatment, while the changes in *Tfap2a* mRNA were delayed until a later time point. This may indicate that *Tfap2a* is regulated at the protein level. Considering that a rapid reduction of the active form of a regulator (here within 6 h) can only be achieved by post-translational mechanisms such as phosphorylation and/or targeted protein decay, the delayed response at the mRNA level appears coherent [42, 43]. Consistent with the changes observed at mRNA level, TFAP2A protein levels tend to decrease in the first 72 h after the TGFβ1 treatment (Fig. 2e and f).

**Fig. 3:**
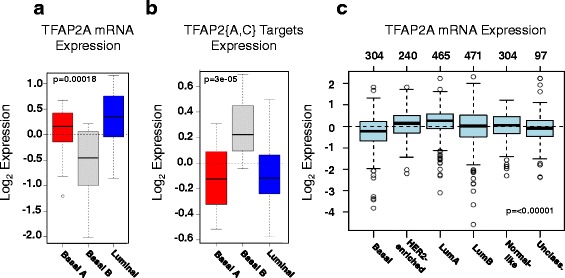
TFAP2A expression and activity in breast cancers. Box plots of TFAP2A gene expression (**a**) and expression levels of the top 100 ISMARA-inferred TFAP2A targets (**b**) in a panel of breast cancer cell lines grouped in the basal A (*red*), basal B (*grey*) and luminal (*blue*) subgroups based on the annotation from Neve et al. [32]. **c** Box plot of TFAP2A gene expression for tumor samples stratified according to PAM50 subtypes [57]. All plots were generated with the GOBO online tool [33]

To gain further insight into the relationship between TFAP2A expression and activity, we examined the mRNA expression data that was previously generated from human breast cancer cell lines [32]. The Neve et al. data set contained 51 samples that were separated in three categories according to their transcriptomic signature. Using the GOBO online tool we found that TFAP2A expression is reduced in the basal B breast cancer cell lines (Fig. 3a), which have a higher expression of the mesenchymal markers compared to the basal A type cell lines (Additional file 1: Figure S6). This is consistent with our observations in the mouse cell line [33]. We also analyzed the Neve et al. dataset [32] in ISMARA to identify the most significant TFAP2{A,C} targets, based on their ISMARA-provided z-score. Using the top 100 TFAP2{A,C} targets as input for the GOBO tool, we found that their expression is significantly increased in the basal B sub-type (Fig. 3b). Thus, we found a strikingly consistent negative correlation between TFAP2A mRNA and the expression of its transcriptional targets in the Neve et al. dataset, as well as in the data that we obtained in the NMuMG model. Remarkably, in a large panel of breast tumor datasets originating from more than 1500 patients, the expression of TFAP2A mRNA is also downregulated in the basal sub-type cancer category (Fig. 3c) [33]. More generally, using mRNA expression data from The Cancer Genome Atlas, we found that the expression of TFAP2A is positively correlated with that of epithelial markers and negatively correlated with that of mesenchymal markers, in normal breast tissue samples as well as in samples from breast tumors (Additional file 1: Figure S7).

### TFAP2A binds directly to the Zeb2 promoter region

**Fig. 4:**
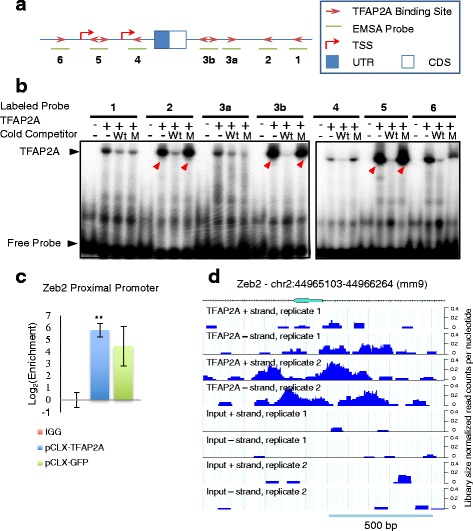
TFAP2A binds directly to the Zeb2 promoter region. **a** Sketch of the region around the second exon of mouse *Zeb2*, showing the two transcription start sites found in SwissRegulon [44]. The *blue filled box* indicates the non-coding untranslated region (UTR) in exon 2, while the *white filled box* designates the start of the coding region (CDS). The predicted TFAP2A binding sites from SwissRegulon are marked with *red arrows*, and the probes that were used in (**b**) are indicated with *green lines* below the gene structure. Predicted transcription start sites (TSS) are also indicated. **b** Radiography of TFAP2A Electrophoretic Mobility Shift Assay (EMSA) with radiolabeled oligonucleotides, each spanning one of the predicted binding sites. The presence or absence of TFAP2A protein in the assay is indicated by a + or – sign, respectively. Cold competitors were used at 200-fold excess over the radiolabelled probes. Wt corresponds to unlabeled probe; M indicates a double-stranded oligonucleotide with a mutated TFAP2A binding site. *Red arrows* indicate the predicted TFAP2A binding probes that behave as expected from specific binding of TFAP2A. **c** TFAP2A ChIP was performed in NMuMG cells stably transduced with pCLX-TFAP2A (denoted as TFAP2A-OE (*blue*)) or with pCLX-GFP (denoted as TFAP2A-GFP (*green*)) viral vectors and further treated with 2 μg/mL doxycycline. Quantitative PCR data shows the enrichment of *Zeb2* promoter relative to a non-transcribed genomic region in TFAP2A-ChIP normalized to IgG control (*red*). Two independent experiments were performed for each condition and shown are means and standard deviations. The one-tail paired *t*-test indicates that TFAP2A is significantly enriched at the *Zeb2* (** for *p* < 0.01). **d** ChIP-seq libraries from TFAP2A ChIP or input chromatin were generated and the coverage of the genomic region spanning the second exon of *Zeb2* by reads is shown in a mouse genome browser (www.clipz.unibas.ch and [45]). The results of two independent experiments are presented. The TFAP2A ChIP-seq the *Zeb2* promoter region previously assessed by qPCR is enriched with respect to the input control sample. Mapping, annotation and visualization of deep-sequencing data was done with the ClipZ server [45]

In addition to the significant activity change of the TFAP2{A,C} motif activity in human and mouse EMT systems (Fig. 1a and b), the interaction of the TFAP2{A,C} and ZEB1,2 motifs was also conserved in the EMT networks of both species. Our analysis predicted that TFAP2{A,C} controls the expression of *ZEB1* and *ZEB2* genes in both systems. The *Zeb2* target has a higher score than *Zeb1* in NMuMG cells (target scores from the initial ISMARA analysis were 0.7 for ZEB1 and 0.51 for ZEB2 in human, and 0.18 and 0.52, respectively in mouse). To validate the interaction between TFAP2A and the *Zeb2* promoter we performed an Electrophoretic Mobility Shift Assay (EMSA). From the SwissRegulon database of transcription factor binding sites that were predicted based on evolutionary conservation (www.swissregulon.ch), we found that the region around the second exon of the *Zeb2* gene, in which the ATG start codon resides, contains seven clusters of consensus binding sites for TFAP2{A,C} with a relatively high posterior probability. The corresponding region is represented in Fig. 4a. Two transcription start sites (TSS), annotated in the SwissRegulon, based on cap analysis of gene expression (CAGE) data [44], are in close proximity to the TFAP2{A,C} binding sites, in the intronic region between the first and the second exon (Fig. 4a) [44]. To confirm that the TFAP2A TF binds to the predicted sites, we carried out EMSA with radiolabeled oligonucleotides, each spanning one of the predicted binding sites (Fig. 4a and b). In the presence of the broad competitor poly-dI-dC, most of the probes give a shift upon addition of TFAP2A. The addition of an excessive amount of cold probes containing the same binding sites (Wt), results in a reduction of the shifted radiolabelled oligonucleotides, indicating competition for specific binding. This is further demonstrated by the fact that only few probes, indicated with red arrows, restored their shift in the presence of cold competitors that contained mutated versions of TFAP2A binding sites (M) (Fig. 4b).

To validate this regulatory interaction in NMuMG cells we have generated a stable cell line in which the overexpression of TFAP2A can be induced with doxycycline (see Methods; Additional file 1: Figure S2). As a control we established a similar cell line using an expression construct in which the TFAP2A coding region (CDR) was replaced by green fluorescent protein (GFP) CDR. Using an antibody that recognizes the endogenous TF we further confirmed that TFAP2A binds to the *Zeb2* promoter region by TFAP2A-chromatin immunoprecipitation (ChIP) followed by quantitative PCR: the *Zeb2* promoter was significantly enriched in the TFAP2A-ChIP from cell lines expressing either exogenously-encoded TFAP2A (*p* = 0.005). Cells expressing only endogenous TFAP2A also showed an enrichment of the the *Zeb2*, albeit not to the same level of significance (*p* = 0.06) (Fig. 4c).

Visualization of ChIP-seq data that we also obtained in this system, with the CLIPZ genome browser (www.clipz.unibas.ch) [45], confirms the presence of a peak in the predicted binding region that is only present in the TFAP2A-ChIP sample, but not in the Input controls (Fig. 4d) or the IgG (not shown). Overall, these results confirm that TFAP2A directly interacts with the *Zeb2* promoter, both in vitro as well as in the NMuMG cell line.

### TFAP2A overexpression in NMuMG modulates epithelial plasticity

**Fig. 5:**
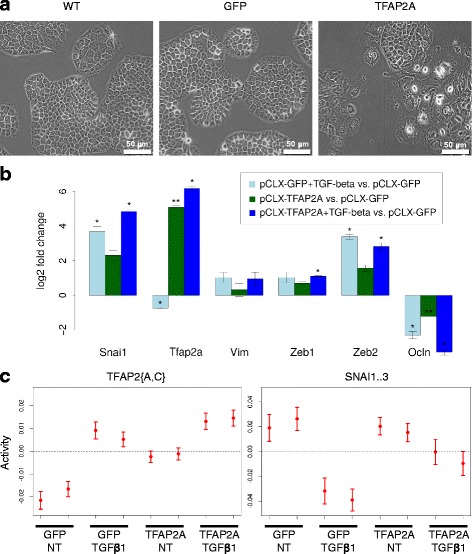
TFAP2A overexpression in NMuMG modulates epithelial plasticity. **a** Expression of either GFP or TFAP2A was induced by 72 h doxycycline treatment in NMuMG cells stably transduced with either pCLX-GFP or pCLX-TFAP2A. Morphological changes and sparse cell arrangement are visible in phase contrast microscopy upon TFAP2A expression. Scale bar: 50 μm. **b** Gene expression log_2_ fold changes of EMT markers (TFs) were calculated from mRNA-seq samples of doxycycline-induced, TGFβ1-treated (72 h, 2 ng/mL) pCLX-GFP (pCLX-GFP + TGF-beta), doxycycline-induced pCLX-TFAP2A (pCLX-TFAP2A), as well as of doxycycline-induced, TGFβ1-treated (72 h, 2 ng/mL) pCLX-TFAP2A (pCLX-TFAP2A + TGF-beta) cell lines relative to doxycycline-induced pCLX-GFP (pCLX-GFP) cell line. Shown are the mean log_2_ fold changes (+/- 1 standard deviation) from two experiments. TFAP2A overexpression is apparent in both TFAP2A-induced samples (*dark green* and *dark blue*) but is not induced in cells treated with TGFβ1 alone (*light blue*). The EMT-inducing TFs have increased expression upon TFAP2A induction. * indicates a *p*-value ≤ 0.05 and ** a *p*-value ≤ 0.01 in a two-tailed *t*-test. **c** The transcriptional activities of TFAP2{A,C} and SNAI1..3 motifs in different conditions, as inferred with ISMARA from mRNA-seq data as described in (**b**). The two replicates from each condition are plotted next to each other

Finally, we used the above-mentioned cell lines to investigate the consequences of perturbed TFAP2A expression. Induced expression of TFAP2A, but not GFP, in untreated NMuMG cells led to morphological changes visible in phase contrast microscopy (Fig. 5a); compared to GFP-expressing cells, TFAP2A-expressing cells lose their epithelial polygonal cell shape and disperse on the plate. Consistently, qRT-PCR showed that adhesion-related genes were specifically deregulated upon TFAP2A induction (Additional file 1: Figure S3a and S3b). As expected, the treatment of GFP-expressing cells with TGFβ1 for 3 days leads to the induction of EMT markers *Snai1*, *Zeb2* and *Vim*. The expression of endogenous *Tfap2a* decreases upon the treatment of GFP-expressing NMuMG cells with TGFβ1. However, the induction of TFAP2A expression in the absence of TGFβ1 treatment appears to promote the expression of core EMT TFs such as *Snai1*, and *Zeb2* (Fig. 5b and Additional file 1: Figure S3c), without affecting the expression of E-cadherin at the mRNA level (Additional file 1: Figure S3a).

To better understand the effect of TFAP2A overexpression, we carried out transcriptional profiling of these four cell populations, namely untreated and TGFβ1-treated GFP-expressing cells, and untreated and TGFβ1-treated (for 72 h) TFAP2A overexpressing cells. The *Tfap2a* expression is increased upon doxycycline induction (Fig. 5b), but it decreases upon TGFβ1 treatment of GFP-expressing control cells (as we have observed before). Notably, the MARA analysis of these data reveals an increased activity of the TFAP2{A, C} motif in TGFβ1-induced, GFP-expressing cells, as we have initially observed in wild-type NMuMG cells, but also in TFAP2A-overexpressing cells treated with the growth factor when compared to GFP-expressing cells (Fig. 5c). The TGFβ1 treatment of TFAP2A-overexpressing cells further increases the TFAP2A activity. Thus, the exogenously introduced TFAP2A has an opposite transcriptional activity relative to the endogenous form.

The activity of the SNAI1 motif decreases upon TGFβ1 treatment while its mRNA level increases, as expected from its known repressive activity in mesenchymal cells [41] (compare Fig. 5b and c). However, the >4-fold increase in *Snai1* mRNA that occurred upon TFAP2A overexpression was followed only by a small decrease in SNAI1 motif activity. Interestingly, the TGFβ1-induced decrease of SNAI1 activity is less pronounced when the TGFβ1 treatment is carried out in TFAP2A-overexpressing cells (Fig. 5b and c). These results indicate that overexpression of TFAP2A perturbs the course of TGFβ1-induced EMT in NMuMG cells.

## Discussion

Metastasis is the leading cause of death among breast cancer patients and a deeper understanding of the process is necessary for the development of treatment strategies [46]. The development of malignancy has been related to epithelial plasticity, and unsurprisingly, regulatory modules and networks that are involved in normal human development are hijacked during tumorigenic processes [41]. Although the regulatory network behind EMT has been intensely studied, by integrating data from multiple systems, recently developed computational methods can continue to provide new insights. In this study we have compared data from both developmental processes and cancer models of epithelial plasticity aiming to identify key regulators that are evolutionarily conserved. We found only a small number of motifs that have a significant activity change upon EMT in both human and mouse systems. Of these, SNAI1..3 and ZEB1..2 correspond to TFs that form the core EMT network [35]. We did not explicitly recover motifs for GSC, TWIST and FOXC2/SLUG. However, only the last factor has a specific motif represented in ISMARA. Motifs for miR-200 and the TGFβ1-related TGFI1 were only identified from the human samples. A novel insight derived from our analysis was that the motif corresponding to the TFAP2A and/or TFAP2C TFs also has a significant contribution to the expression changes that occur upon EMT in both species (Fig. 1a and b). The mechanistic link between TFAP2A/C and EMT was so far unknown, although TFAP2A was previously found important for neural crest formation and implicated in the activation of EMT inducing factors [47]. Furthermore, TFAP2A and TFAP2C have been implicated in mammary gland tumorigenesis and metastasis formation [16, 19]. Our data demonstrates that TFAP2A activity dynamically changes in the early time points of the TGFβ1 induced EMT in NMuMG cells, and thus suggests that TFAP2A regulates early steps in this process (Fig. 2c). Although our analysis of the EMT time series indicated that the expression of *Tfap2a* is negatively correlated with the expression of its targets (reflected in the motif activity, Additional file 1: Figure S4), overexpression of TFAP2A induces changes that are similar to those occurring upon *Tfap2a* downregulation during EMT. This observation can have multiple causes. One is that TFAP2A activity is regulated post-translationally, similar to the core EMT TFs [41]. For instance, the SNAI1 protein has a rapid turn-over and its stability and activity are regulated by post-translational phosphorylation, lysine oxidation and ubiquitylation [41]. Indeed, it has been demonstrated that the sumoylation and phosphorylation of the TFAP2A protein can affect its transcription activation or DNA binding functions [48, 49]. Therefore, it is possible that during EMT, the activity of TFAP2A on its targets changes from repressive to activating and its mRNA levels may decrease due to a feedback regulatory mechanism. A regulatory step at the protein level is also suggested by the fact that the highest increase in TFAP2A activity is observed in the first 6 h of treatment whereas the changes in the *Tfap2a* mRNA are delayed to a later time point (Fig. 2c and d). Alternatively, TFAP2A may activate some of its targets and repress others, so that which effect dominates overall will depend on other factors or on TFAP2A expression levels. The dual transcription activity of TFAP2A has also been reported before [16]. Yet another possibility is that depending on its mode of expression and of post-translational modifications, TFAP2A may form distinct complexes with other factors to activate or repress its targets. Additional experiments will be necessary to address these possibilities. Nevertheless, our data provides evidence for a direct regulatory link between TFAP2A/C and the core EMT regulators ZEB1 and ZEB2 in both human and mouse. In mouse, we found that TFAP2A binds to the *Zeb2* promoter (Fig. 4), and that *Zeb2* levels increase when TFAP2A is overexpressed (Fig. 5b). These results indicate that TFAP2A regulates EMT-inducing factors transcriptionally. Although we have not investigated it in detail here, our TFAP2A-ChIP-seq data suggests that other critical regulators of EMT such as *Snai1*, *Sox4*, *Ezh2* and *Esrp2* may also be targets of TFAP2A (Additional file 1: Figure S5). This further strengthens the hypothesis that TFAP2A is part of a densely-connected network of genes that are essential for EMT [50–52]. Consistent with exogenous TFAP2A-induced activation of EMT markers, the NMuMG cells that overexpressed TFAP2A underwent phenotypical changes that were indicative of the acquisition of a mesenchymal phenotype (Fig. 5a). Furthermore, an EMT signature of positively regulated genes was significantly represented among genes that were up-regulated in TFAP2A-overexpressing NMuMG cells compared to control, GFP-expressing cells (Additional file 1: Table S4) [35]. Genes involved in cellular adhesion and glycosphingolipid metabolism, which has been recently suggested to regulate cellular adhesion via *St3gal5* and, more upstream, *Zeb1* [53], seems to also be affected by TFAP2A overexpression (Fig. 5b; Additional file 1: Figure S3b and S3c). Cell adhesion is concomitantly affected (Fig. 5a). Thus, our results support the link between TFAP2A and ZEB TFs, although overexpression of TFAP2A leads to cellular that are observed upon TGFβ1-induced down-regulation of endogenous TFAP2A. One cannot exclude that the observed induction of an EMT response upon TFAP2A overexpression is due to a phenomenon similar to the so-called ‘squelching effect’ [54]. The activity of TFAP2A does not appear to be sufficient for the induction of a complete EMT phenotype in the absence of TGFβ1 (Fig. 5a, c). Previously, ChIP-chip-based measurements of SMAD2/3 binding in human keratinocytes upon TGFβ stimulation indicated that SMAD2/3 binding sites co-occur with those for TFAP2A/C TFs, leading to the hypothesis that TFAP2A is involved in mediating the TGFβ signaling [55]. However, maintaining a high TFAP2A level in the context of TGFβ signaling may interfere with the activity of EMT TFs (Fig. 5c), consistent with our observation that EMT factors such as SNAI1 have less repressive activity when TFAP2A is overexpressed during TGFβ1-induced EMT. This in turn could be the rationale for the moderate downregulation in *Tfap2a* levels that we observed in the later phases of the TGFβ1-induced EMT time course (Fig. 2c). Consistent with previous studies that suggested that TFAP2A activation is connected with the luminal breast phenotype, thus promoting the epithelial state [16], here we found that endogenously-encoded TFAP2A is down-regulated upon TGFβ1-induced EMT. Interestingly, PRRX1, another TF that promotes EMT in a developmental context, was found to both induce the transition, and reduce the metastatic potential in tumors [56]. This suggests that the two processes are not always coupled and that a tumor suppressor can also activate EMT. This may be the case with TFAP2A as well; while it mediates the initiation of EMT, its sustained expression may interfere with EMT signaling. Our data thus connects TFAP2A to the core regulatory network that orchestrates the epithelium-to-mesenchyme transition in normal development as well as in cancers.

## Conclusions

Applying recently developed computational methods to a set of epithelial plasticity datasets we have construct a conserved transcription factor motif interaction network that operates during the epithelium-to-mesenchyme transition. Our analysis recovered the known core EMT TFs and further linked the TFAP2A/C motif to this core network. Employing the NMuMG model cell line we provided further evidence that TFAP2A is involved in EMT, most likely in the early stages. We found that TFAP2A binds to the promoter of the Zeb2 master regulator of EMT and that TFAP2A overexpression in NMuMG cells induces an increase in Zeb2 expression. Finally overexpression of TFAP2A in NMuMG cells promoted the expression of EMT markers and of cellular features related to the acquisition of a mesenchymal phenotype. Overall, our data links TFAP2A to the core TF network that is regulating EMT in normal development as well as in cancers.

## Reviewers’ comments

### Reviewer’s report 1: Dr. Martijn Huynen, Nijmegen Centre for Molecular Life Science, The Netherlands

## Reviewer comments

The manuscript describes an elegant computational analysis of the regulatory motifs associated with the EMT transition, followed by the experimental validation that a new factor, TFAP2A, plays an important role in this process. In general I do find the first part of the paper very convincing, it computationally identifies the factor, confirms the results in independent data, and confirms binding of the factor to a predicted target. I do get a bit confused by the results of the overexpression of TFAP2A, and the arguments used to make these results consistent with the first part of the paper.

Author’s response: *We thank the reviewer for the positive assessment of our computational analysis. Although we did find publicly available data that supports our conclusions about the involvement of TFAP2A in EMT, we nevertheless sought to validate its role ourselves. We tried to explain better the rationale and the results in the revision, even though some results remain paradoxical.*

Does Fig. 1 contain the complete set of motifs that are predicted to be "differentially active" in the transition? If so, is it a coincidence that they are all connected to each other?

Author’s response: *We have described the selection of the motifs that we show in the legend of the Figure. Briefly, we only showed motifs with an absolute z-score > 2 and arrows that represent predictions with probabilities larger than a threshold (0.35 for human and 0.15 for mouse). For the readability of Fig.* 1 *, only motifs that have at least a predicted interaction with another motif at the mentioned thresholds are considered. However, realizing that motifs with significant activity that are not connected to other motifs may also be of interest, we have now included the full tables of motif activity changes upon EMT as Additional file* 1 *: Tables S2 and S3.*

I am surprised by the low level of conservation between the species. Are there some motifs from e.g. human that are just below a threshold? The authors argue "The motif interaction networks derived from mouse and human EMT models suggest that only a small fraction of the TFs has a highly conserved and significant role in both species." How reliable are those species-specific predictions, and how reliable is the absence of a signal in these analyses, with these data.

Author’s response: *Although we selected sequencing data sets obtained from systems where EMT presumably occurs for both species, we unfortunately did not have matching systems available for human and mouse. So indeed, the precise scores of the different motifs depend on the data sets that we used and given sufficient data, other motifs may emerge as having similar behaviour in mouse and human EMT systems. Nevertheless, we found it reassuring that the core EMT factors that were extensively studied so far, such as SNAI and ZEB emerged from our analysis. That the TFAP2A,C motif also has a conserved function was unexpected and prompted us to study it further.*

If I understand the manuscript correctly, the downregulation of TFAP2A is associated with the epithelial to mesenchymal transition. Why then overexpress TFAP2A? Even is this has to do with technical limitations, I would like to see that mentioned explicitly to better understand the logic of the approach.

Author’s response: *Our initial analysis indicated that the expression of TFAP2A is down-regulated during EMT (Fig.* 2 *), while its motif activity increases, suggesting that TFAP2A may function as a repressor. Therefore, we overexpressed TFAP2A, reasoning that this should perturb the process of TGFb1-induced EMT. Indeed, this is also what we observe. However, analysis of the sequencing data obtained after TFAP2A overexpressionoverexpression also revealed some paradoxical results, which we addressed in our discussion.*

I find the discussion why "overexpression of TFAP2A induces changes that are similar to those occurring upon Tfap2a downregulation during EMT" lengthy and unconvincing. The authors first perform a very thorough quantitative analysis of gene expression and motif occurrence data, based on the simplifying but defendable assumptions of their linear model, confirm their findings in independent breast cancer data (Fig. 3). Then they use a large number of ad-hoc arguments to explain the inconsistencies in their results. They may all be true, but they are not convincing. Given the apparent contradictory results of the overexpression, I am surprised by the sentence "Finally, we confirm that overexpression of TFAP2A in NMuMG cells modulates epithelial plasticity and cell adhesion" in the abstract as those results do not confirm a specific hypothesis based on the results of the quantitative analysis.

Author’s response: *We have revised the discussion to hopefully make it more streamlined. We agree with the reviewer that the initial computational analysis suggested a clear picture of TFAP2’s involvement in EMT. However, as we tried to go deeper into the mouse model, the results that we obtained were more complex than we anticipated. We felt it was important to show the unexpected overexpression results, but in the revision we have included only the initial characterization of this cell line, without following it into the phenotypic analysis. We hope that our revised description of the results makes it clear what we have learned from the different systems about the behaviour and role of TFAP2A.*

In Fig. 5c there is a line connecting the various constructs. I take it this is not meant to implicate some sort of continuity? I do fully support publication once these issues have been handled.

Author’s response: *Thank you for pointing this out. We have removed the lines to prevent the illusion of continuity of the data points.*

editorial: The legend with Fig. 3 could use some work "ABasal" or "Basal A"?

Author’s response: *We thank the reviewer for pointing this out. We have fixed this issue and made the labels easier to read.*

TFAP2A expression was found to be less organized in breast cancer compared to normal mammary gland. - > glands

Author’s response: *We think that the original formulation is correct.*

what is "substantially expressed"

Author’s response: *We have explained that only Tfap2a (and not the other family members) has read coverage in all exons.*

It would be nice to specify which TFs of the core EMT network of ref 33 are retrieved and which are not.

Author’s response: *We have expanded the text accordingly.*

"transcriptional" can often be replaced by "transcription", e.g. in "transcriptional regulation" page 18,

Author’s response: *We have changed the term in all places where we thought it makes sense.*

line 20 "the interactions of the TFAP2{A,C}" appears redundant.

Author’s response: *We removed the redundancy.*

page 22. "in untreated NMuMG cells lead to morphological changes" -- > "led"

Author’s response: *Fixed.*

"an EMT signature of positively regulated genes were significantly represented" -- > "was"

Author’s response: *Fixed.*

### Reviewer’s report 1: Dr. Nicola Aceto, Department of Biomedicine, University of Basel, Switzerland

## Reviewer comments

Dimitrova et al. present a manuscript in which they highlight the transcription factor TFAP2A as a novel EMT regulator. They suggest that TFAP2A target genes, such as ZEB2, are upregulated during EMT in the NMuMG mouse model. Further, they conclude that the interaction between TFAP2A and ZEB2 promoter affects ZEB2 expression, hence modulating the EMT process itself and providing evidence for a role of TFAP2A in cancer progression. Altogether, this is an interesting manuscript yet requiring a few modifications and clarifications to convincingly argue in favor of TFAP2A’s role in cancer progression.

(1) Introduction: the authors write their introductory paragraph arguing that e.g. “cancer progression, metastasis and chemotherapy resistance have all been linked to EMT”. However, the role of EMT for each of these processes is highly debated in the field, and I would suggest the authors to provide a more balanced introduction, where it is clearly stated (and referenced) that the role/requirement of EMT in all these processed has still to be fully understood, especially in clinically-relevant settings.

Author’s response: *We have rephrased and provided additional references to make the introduction more balanced.*

(2) Fig. 2a: I remain unconvinced about the degree of EMT that is triggered by TGFb in NMuMG cells. For instance, why only a small fraction of control cells express E-cad (roughly 30%)? Looking at the TGFb-treated cells, this ratio appears to remain the same (3/9 cells, i.e. roughly 30%). TFAP2A-positive vs negative cells in control vs TGFb also do not seem to change much, and neither does actin. I would suggest the authors to provide more quantitative data here (% of positive cells for each marker, or signal intensity) that comprise several fields of view.

Author’s response: *To answer the reviewer’s questions, we have redone the experiment, and imaged the cells with higher magnification. The results in the revised Fig.* 2 *clearly show that TFAP2A is abundantly expressed and nuclearly localized in control cells, while this staining pattern is abrogated upon TGFb1 treatment. In almost all control cells, the expression of E-cadherin is clearly visible, as is its localization close to the plasma membrane, features which are also abrogated by the TGFb1 treatment. E-cadherin levels estimated by Western blot (Fig.* 2 *e) also indicate down-regulation upon TGFb1 treatment.*

(3) Fig. 2c: how relevant is a Z-value of 3, with an activity range varying from -0.02 to 0.01? Looking at Fig. 1 (Z-values ranging from -19 to +19), can the authors convincingly state that TFAP2 target genes (and TFAP2 activity, respectively) significantly change upon TGFb treatment in NMuMG cells?

Author’s response: *Please note that Fig.* 1 *was generated based on multiple data sets and that is why the z-scores cover a much larger range. Based on a standard normal distribution of z-scores we consider values larger than 2 (in absolute value) significant.*

(4) Fig. 2d: somehow related to the previous point. Changes in TFAP2A protein levels are not very impressive. Is the change statistically significant? Control does not seem to have any error bar, was it repeated more than once?

Author’s response: *We have repeated this experiment as well, using three biological replicates, adding an additional control (actin, in addition to lamin and GAPDH) and also Ponceau staining (current Fig.* 2 *e). Although the overall protein levels are similar between conditions, TFAP2A’s expression decreases upon TGFb1 treatment (as apparent also from the immunofluorescence staining, Fig.* 2 *a). The controls that we initially used, lamin and GAPDH, also decrease to some extent upon TGFb1 treatment, which is probably why the relative change in TFAP2A in our initial figure was not very impressive. However, relative to the total protein level as well as to actin, TFAP2A expression is clearly reduced by the TGFb1 treatment.*

(5) Fig. 3: The authors observe a correlation between low TFAP2A expression and basal type of breast cancer. Two questions arise here: (a) is basalB more EMT-like than basal-A?

Author’s response: *In the original publication (Ringner* et al. *PLoS One, 6:e17911, 2011), the basal B type is considered “more stem like”.*

(b) how are TFAP2A target genes behaving in the larger dataset with 1500 samples?

Author’s response: *Unfortunately we could not carry out this analysis on the GOBO web server.*

(6) Fig. 5: could the authors elaborate more about their conclusion “TFAP2A perturbs the course of TGFb-induced EMT in NMuMG cells”? It seems here that TFAP2A mRNA expression and activity are somewhat disconnected here, yet in previous experiments they seem to be going along quite well (e.g. see Fig. 2b-c and Fig. 3).

Author’s response: *The reviewer, as reviewer #1 as well, rightly points out that the TFAP2A that is expressed from the exogenous construct seems to behave differently than the endogenously-encoded gene. This is also apparent from the quantification of TFAP2A expression in TGFb1-treated control cells, that only express endogenously encoded TFAP2A (which is down-regulated by the treatment) and in TFAP2A overexpressionoverexpression (where the expression is up-regulated, as expected, Fig.* 5 *b). We discuss possible causes for this discrepancy in our manuscript (* Discussion *section). Although we did not identify the precise cause for it, we felt that it was important to show these results.*

(7) Fig. 6: In some instances (i.e. in TGFb-treated samples), actin staining seems to extend to regions that do not display any Hoechst staining. For example, in TFAP2A + TGFb sample, actin staining shows cells on the lower right corner of the image, but those cells do not show up in the Hoechst staining.

Author’s response: *We think that this had to do with the intensity of the signal. However, we removed this figure from the revised version of the manuscript.*

(8) Differences in the aggregation index are not very impressive, and when taken per se would not be a strong argument of the involvement of TFAP2A in EMT. Instead, what would be the effect -in terms of EMT genes expression- of depleting TFAP2A in NMuMG cells treated with TGFb?

Author’s response: *Because endogenous TFAP2A is down-regulated upon TGFb1 treatment, we initially sought to perturb the course of EMT by overexpressing TFAP2A and we carried out most of the experiments with this construct. It turned out that the overexpression of TFAP2A leads to similar molecular signatures as the downregulation of endogenous TFAP2A that takes place upon TGFb1-induced EMT. We agree with the reviewer that presenting the results with this construct as well as with the siRNAs makes the interpretation very difficult. We therefore decided to remove this figure and close the study at the point where the exogenous construct showed paradoxical results.*

The authors show in Additional file 1: Figure S3 some EMT genes, but it seems that genes such as Vim and Ocln are missing.

Author’s response: *We have regenerated panel b in Fig.* 5 *based on the mRNA-seq samples that we used to infer the motif activities shown in panel c of the figure and we have included also Ocln, aside from Vim, whose expression we also estimated by qPCR. Both of the markers behave as expected in EMT. The additional qPCR validations are now shown in Additional file* 1 *: Figure S3c.*

Also, what is the TFAP2A knockdown level with the siRNAs?

Author’s response: *As we explained above, because the results of perturbing TFAP2A expression were difficult to interpret, we decided to not pursue too far the perturbation experiments. Therefore, we removed Fig. 6 and we did not include the siRNA quantifications in the revised manuscript.*

(9) Generally, it would be great to show some functional assays related to EMT (e.g. Boyden chamber, etc.) to reinforce the involvement of TFAP2A in this process

Author’s response: *We agree with the reviewer that it would be exciting to carry out these studies. However, as the reviewer probably appreciates, this regulatory network is very complex and the perturbation experiments did not turn out as we expected. We therefore decided to follow the suggestion of reviewer #1, concentrating on the comparative analysis of the different systems that yielded consistent results and not trying to resolve the specific mechanism of TFAP2A, which likely depends on the precise form of the protein that is expressed from the endogenous locus.*

### Reviewer’s report 2: Dr. Martijn Huynen, Nijmegen Centre for Molecular Life Science, The Netherlands

## Reviewer comments

This reviewer provided no additional comments.

### Reviewer’s report 2: Dr. Nicola Aceto, Department of Biomedicine, University of Basel, Switzerland

## Reviewer’s comments

Dimitrova et al. present a revised version of the manuscript that addressed and discussed some of the initial concerns. While I find the manuscript worthy of publication, a few points are still worth mentioning: (1) In an answer to my previous question #5 (see 1st review) the authors argue that Basal B is considered more stem-like (therefore more mesenchymal) than Basal A. However, EMT and stem-like are two very different features of cancer cells as well as normal tissues, which may or may not overlap depending on a variety of factors. For instance, a number of tumor cell lines that are fully epithelial can display stem-like features (tumor initiation, self-renewal, differentiation). My original question was more whether by looking at gene expression data of Basal B, this tumor type expresses significantly more EMT markers than Basal A. This would reinforce their conclusions.

Author’s response: *To answer the reviewer’s question we have used the GOBO tool to compare the expression levels of various epithelial and mesenchymal markers in Basal A and Basal B tumor types. As shown in the new Additional file* 1 *: Figure S6, epithelial markers have higher expression in Basal A tumors, whereas mesenchymal markers have higher expression in Basal B tumors. This is in line with the concept that Basal B tumors are more mesenchymal.*

(2) Regarding patient data it would be more convincing to check the expression of TFAP2 (as well as its target genes and EMT markers) in several independent datasets to reinforce the conclusions of the authors.

Author’s response: *To answer the reviewer’s second question, we have used yet another data set, namely expression profiles of tumors and normal tissue samples from The Cancer Genome Atlas, to further examine the relationship between the expression of TFAP2A and that of various epithelial and mesenchymal markers. These results, summarized in the new Additional file* 1 *: Figure S7, show that the TFAP2A expression is positively correlated with that of epithelial markers and negatively correlated with that of mesenchymal markers. This is again consistent with the results we obtained in our experimental system (Fig.* 2 *).*

## Additional file

Additional file 1: Supplementary information. (DOCX 19403 kb)

## Abbreviations

BOFS: Branchio-Oculo-Facial Syndro
CAGE: Cap analysis of gene expression
CDR: Coding region
ChIP: Chromatin immunoprecipitation
EMT: Epithelial to mesenchymal transition
ESC: Embryonic stem cells
GOBO: Gene expression-based outcome for breast cancer online
MARA: Motif-activity response analysis
NC: Neural crest
NCBI: National Center for Biotechnology Information
NMuMG: Mouse mammary gland epithelial cell line
SELEX: Systemic evolution of Ligand by EXponential enrichment
SRA: Sequence read archive
TF: Transcription factor
WB: Western blot
