# Supplementary material for: TFAP2A is a component of the ZEB1/2 network that regulates TGFB1-induced epithelial to mesenchymal transition
Source: Biol Direct. 2017 Apr 17;12:8. doi: 10.1186/s13062-017-0180-7 (PMC5392957; doi:10.1186/s13062-017-0180-7)
Supplement: Additional file 1: — Supplementary information. (DOCX 19403 kb) [file 13062_2017_180_MOESM1_ESM.docx]

**Supplementary Information**

**TFAP2A is a component of the ZEB1/2 network that regulates TGFB1-induced epithelial to mesenchymal transition**

Yoana Dimitrova^1^, Andreas J. Gruber^1^, Nitish Mittal^1^, Souvik Ghosh^1^, Beatrice Dimitriades^1^, Daniel Mathow^3^, William Aaron Grandy^1^, Gerhard Christofori^2^, Mihaela Zavolan^1,4^

^1^Biozentrum, University of Basel, Klingelbergstrasse 50-70, CH-4056 Basel, Switzerland.

^2^Department of Biomedicine, University of Basel, Mattenstrasse 28, CH-4058 Basel, Switzerland

^3^Department of Cellular and Molecular Pathology, German Cancer Research Center (DKFZ), Heidelberg, Germany.

^4^Correspondence should be addressed to Mihaela Zavolan Biozentrum, University of Basel, Klingelberstrasse 50-70, CH-4056 Basel, Switzerland [mihaela.zavolan@unibas.ch](mailto:mihaela.zavolan@unibas.ch).

**Table-S1. Datasets used for the generation of Figure 1.**

| Accession | Species | Description | Reference |
| --- | --- | --- | --- |
| GSE44727 | human | iPS cells vs iPS derived neural crest cells | [^1^](#_ENREF_1) |
| GSE23833 | mouse | NM18 cells transfected with 25nm scrambled siRNA for 48hrs vs transfected with 25nm scrambled siRNA for 48hrs and treated with TGFβ1 for 40hrs | [^2^](#_ENREF_2) |
| GSE49151 | mouse | NMuMG/E9 cells treated with Control siRNA 1 or Control siRNA 1 and TGFβ1 | [^3^](#_ENREF_3) |
| GSE21668 | human | Undifferentiated embryonic stem cells, H9 vs mesodermal progenitor population | [^4^](#_ENREF_4) |
| GSE9691 | human | HMLE cells untreated vs TGFβ1 treated | [^5^](#_ENREF_5) |
| GSE55711 | mouse | Py2T untreated vs  Py2T 5 days TGFβ | [^6^](#_ENREF_6) |
| GSE55964 | mouse | Neuroepithelim vs neural crest | [^7^](#_ENREF_7) |

**Table-S2. Motif activity changes derived from human EMT models (sorted by absolute z-scores).**

| **Motif** | **Z-Score** |
| --- | --- |
| SNAI1..3.p2 | -24.288498 |
| ZEB1.p2 | -15.499399 |
| TFAP2{A,C}.p2 | 13.229919 |
| bHLH_family.p2 | -9.973254 |
| ARNT_ARNT2_BHLHB2_MAX_MYC_USF1.p2 | -9.771872 |
| ATF6.p2 | 9.291562 |
| HBP1_HMGB_SSRP1_UBTF.p2 | 9.226859 |
| MAFB.p2 | 9.049806 |
| UCCAGUU | 8.346224 |
| TGIF1.p2 | 8.153858 |
| AAUACUG | 8.008944 |
| GTF2I.p2 | 7.767217 |
| ZNF238.p2 | 7.406887 |
| TFEB.p2 | 7.375766 |
| GAGGUAG | 6.920853 |
| NR4A2.p2 | -6.870112 |
| IKZF2.p2 | 6.531350 |
| ESRRA.p2 | -6.402536 |
| ZNF384.p2 | 6.350658 |
| XBP1.p3 | 6.286392 |
| FOX{I1,J2}.p2 | 6.271248 |
| HNF4A_NR2F1,2.p2 | -6.229455 |
| ZNF423.p2 | -6.162108 |
| HOX{A6,A7,B6,B7}.p2 | 6.099096 |
| SREBF1,2.p2 | 6.086621 |
| CUCCCAA | -5.966596 |
| KLF12.p2 | 5.946872 |
| RFX1..5_RFXANK_RFXAP.p2 | 5.541792 |
| CRX.p2 | -5.209987 |
| TEAD1.p2 | 5.085874 |
| MYOD1.p2 | -5.075488 |
| SRF.p3 | 5.044129 |
| GUAAACA | 5.000653 |
| UCACAGU | 4.838087 |
| RXR{A,B,G}.p2 | 4.822125 |
| FOXN1.p2 | 4.817176 |
| TFAP4.p2 | 4.809295 |
| IKZF1.p2 | -4.799092 |
| NKX3-1.p2 | -4.792145 |
| POU5F1_SOX2{dimer}.p2 | -4.723304 |
| NR6A1.p2 | -4.421826 |
| UGUGCUU | -4.412173 |
| DBP.p2 | 4.382192 |
| NFE2L1.p2 | 4.332266 |
| REST.p3 | -4.301219 |
| TFDP1.p2 | 4.187116 |
| PAX5.p2 | -4.168027 |
| FEV.p2 | 4.160352 |
| NFE2L2.p2 | -4.136635 |
| MTF1.p2 | -4.071565 |
| GAUUGUC | 4.043714 |
| PITX1..3.p2 | -4.039921 |
| HLF.p2 | -4.017240 |
| PBX1.p2 | 4.008166 |
| MYBL2.p2 | 3.965618 |
| HAND1,2.p2 | -3.960443 |
| TCF4_dimer.p2 | -3.950222 |
| GGAAUGU | 3.914362 |
| STAT2,4,6.p2 | 3.882966 |
| AACACUG | 3.866572 |
| UUUUUGC | -3.861829 |
| SPIB.p2 | 3.848118 |
| LHX3,4.p2 | -3.825488 |
| GAGAUGA | 3.706771 |
| BPTF.p2 | 3.694402 |
| AAGUGCU | 3.625894 |
| NHLH1,2.p2 | 3.563439 |
| ESR1.p2 | -3.563007 |
| POU6F1.p2 | 3.556815 |
| SRY.p2 | -3.501360 |
| FOX{D1,D2}.p2 | 3.460886 |
| NR1H4.p2 | -3.444054 |
| AGUGGUU | 3.428867 |
| UUGGCAC | 3.428561 |
| POU1F1.p2 | -3.427446 |
| AHR_ARNT_ARNT2.p2 | -3.402815 |
| ACAGUAU | 3.396875 |
| SOX17.p2 | 3.388932 |
| ZIC1..3.p2 | 3.380114 |
| EN1,2.p2 | -3.358870 |
| PRRX1,2.p2 | 3.351237 |
| GATA6.p2 | 3.313266 |
| AGCACCA | 3.258944 |
| AGCAGCG | -3.188248 |
| PAX4.p2 | 3.186388 |
| SOX5.p2 | -3.159007 |
| STAT1,3.p3 | 3.158086 |
| YY1.p2 | -3.150770 |
| NFKB1_REL_RELA.p2 | 3.142962 |
| UUGGCAA | 3.116745 |
| CACAGUG | 3.073683 |
| SPZ1.p2 | 3.050127 |
| NKX2-3_NKX2-5.p2 | -3.014212 |
| TFAP2B.p2 | -2.951623 |
| AGCAGCA | 2.933069 |
| GUAACAG | 2.918261 |
| HOXA9_MEIS1.p2 | -2.883912 |
| ZBTB16.p2 | -2.880101 |
| UCACAUU | -2.879964 |
| CUUUGGU | 2.874260 |
| RBPJ.p2 | -2.843359 |
| UUGGUCC | 2.837260 |
| ETS1,2.p2 | -2.824302 |
| HES1.p2 | -2.801785 |
| MSX1,2.p2 | -2.768325 |
| NRF1.p2 | 2.764340 |
| EHF.p2 | -2.747095 |
| GUAGUGU | 2.740297 |
| AAGGUGC | 2.704792 |
| TBP.p2 | 2.627495 |
| UUGUUCG | 2.612341 |
| ACAUUCA | 2.609181 |
| HNF1A.p2 | -2.574668 |
| RORA.p2 | -2.552431 |
| CDC5L.p2 | -2.543031 |
| UCAAGUA | 2.485514 |
| EP300.p2 | 2.478716 |
| TBX4,5.p2 | 2.452506 |
| SOX{8,9,10}.p2 | 2.443552 |
| CREB1.p2 | -2.429477 |
| JUN.p2 | 2.416194 |
| CEBPA,B_DDIT3.p2 | -2.399835 |
| FOX{F1,F2,J1}.p2 | -2.253559 |
| HIF1A.p2 | -2.245699 |
| GCAGCAU | 2.223872 |
| RXRA_VDR{dimer}.p2 | -2.208193 |
| PATZ1.p2 | -2.199593 |
| GFI1.p2 | 2.060710 |
| ZNF148.p2 | -2.056085 |
| MYB.p2 | 2.050658 |
| UGCAUAG | 2.024762 |
| AGGUAGU | 1.984808 |
| POU3F1..4.p2 | -1.981746 |
| CDX1,2,4.p2 | -1.969056 |
| LEF1_TCF7_TCF7L1,2.p2 | 1.949589 |
| ZBTB6.p2 | -1.941322 |
| FOXP3.p2 | -1.914797 |
| TFCP2.p2 | 1.888318 |
| EOMES.p2 | 1.885703 |
| MEF2{A,B,C,D}.p2 | -1.881713 |
| TAL1_TCF{3,4,12}.p2 | -1.876640 |
| AACAGUC | 1.861035 |
| CAGUGCA | 1.830254 |
| CCUUCAU | 1.823326 |
| NANOG{mouse}.p2 | -1.809389 |
| UCCCUUU | 1.803226 |
| GUGCAAA | 1.799525 |
| BACH2.p2 | -1.778019 |
| GGCUCAG | 1.772541 |
| CTCF.p2 | 1.734880 |
| GGCAAGA | 1.734210 |
| EBF1.p2 | -1.724006 |
| GCUACAU | 1.657170 |
| ZFP161.p2 | 1.629917 |
| NFY{A,B,C}.p2 | 1.620166 |
| HMX1.p2 | -1.605599 |
| FOXL1.p2 | 1.586486 |
| AUGGCUU | 1.583830 |
| CCAGCAU | -1.563745 |
| NFIX.p2 | -1.550922 |
| T.p2 | -1.545058 |
| ONECUT1,2.p2 | -1.463027 |
| AGCUGCC | -1.446600 |
| FOXD3.p2 | -1.313682 |
| NKX2-2,8.p2 | 1.311760 |
| MAZ.p2 | 1.311673 |
| PAX6.p2 | -1.307375 |
| AACCUGG | -1.306978 |
| AUGGCAC | 1.279688 |
| ARID5B.p2 | -1.251386 |
| UAAGACG | 1.223018 |
| CCAGUGU | 1.199163 |
| AIRE.p2 | 1.197368 |
| ZNF143.p2 | -1.163473 |
| ADNP_IRX_SIX_ZHX.p2 | 1.112200 |
| CAGCAGG | -1.112018 |
| AGUGCAA | 1.106508 |
| GCUGGUG | 1.105037 |
| AAUCUCU | -1.096328 |
| DMAP1_NCOR{1,2}_SMARC.p2 | -1.088310 |
| FOXO1,3,4.p2 | 1.083276 |
| UAAGACU | 1.079116 |
| HSF1,2.p2 | 1.069167 |
| ELF1,2,4.p2 | 1.041648 |
| NKX2-1,4.p2 | -1.019541 |
| RXRG_dimer.p3 | 0.998680 |
| STAT5{A,B}.p2 | 0.989426 |
| AR.p2 | -0.987129 |
| HOX{A4,D4}.p2 | -0.963680 |
| ACUGCAU | -0.961381 |
| CGUGUCU | -0.915790 |
| FOXA2.p3 | -0.889308 |
| UAAUGCU | 0.882457 |
| ATF4.p2 | -0.880505 |
| LMO2.p2 | -0.852996 |
| CUACAGU | -0.851979 |
| TLX2.p2 | 0.838461 |
| AUGACAC | -0.836765 |
| NR3C1.p2 | 0.830968 |
| GGAGUGU | -0.802679 |
| ACCCUGU | -0.777607 |
| NFIL3.p2 | -0.769865 |
| ACAGUAC | 0.769100 |
| PAX2.p2 | 0.761529 |
| NFE2.p2 | 0.736988 |
| ALX1.p2 | -0.736058 |
| FOXQ1.p2 | -0.692554 |
| GGAAGAC | 0.671442 |
| CGUACCG | 0.656731 |
| GAUCAGA | 0.656140 |
| PDX1.p2 | 0.650568 |
| HIC1.p2 | 0.635679 |
| EGR1..3.p2 | 0.629637 |
| GGACGGA | -0.612295 |
| ACUGGCC | -0.610809 |
| POU2F1..3.p2 | -0.610074 |
| AAUGCCC | -0.584786 |
| GATA1..3.p2 | 0.581988 |
| NFATC1..3.p2 | 0.581690 |
| SMAD1..7,9.p2 | -0.561780 |
| NKX3-2.p2 | 0.551091 |
| NKX6-1,2.p2 | 0.543763 |
| NR5A1,2.p2 | -0.534370 |
| ATF2.p2 | -0.522030 |
| FOX{C1,C2}.p2 | 0.519247 |
| GZF1.p2 | 0.514649 |
| AGCUUAU | 0.509781 |
| PPARG.p2 | 0.485195 |
| IRF1,2,7.p3 | -0.472821 |
| VSX1,2.p2 | 0.470825 |
| EWSR1-FLI1.p2 | -0.452944 |
| UAUUGCU | -0.435356 |
| E2F1..5.p2 | 0.425908 |
| UGAAAUG | -0.412954 |
| UGCAUUG | -0.406212 |
| AAUCUCA | -0.403747 |
| AUGUGCC | 0.398050 |
| UGUGCGU | -0.380793 |
| GFI1B.p2 | -0.379048 |
| AAAGUGC | -0.378342 |
| SOX2.p2 | -0.378160 |
| ELK1,4_GABP{A,B1}.p3 | 0.340488 |
| GAGAACU | 0.334635 |
| CCCUGAG | 0.306717 |
| GTF2A1,2.p2 | -0.297972 |
| AACGGAA | -0.286985 |
| MZF1.p2 | 0.286773 |
| GLI1..3.p2 | 0.268608 |
| GGCAGUG | -0.258552 |
| GUCAGUU | -0.257585 |
| TP53.p2 | -0.220073 |
| PRDM1.p3 | -0.216004 |
| RREB1.p2 | -0.206545 |
| AACCGUU | -0.202728 |
| AAGGCAC | 0.200079 |
| EVI1.p2 | 0.198591 |
| KLF4.p3 | -0.184265 |
| HOX{A5,B5}.p2 | 0.176179 |
| PAX8.p2 | -0.161384 |
| FOSL2.p2 | 0.138129 |
| POU5F1.p2 | 0.137880 |
| AUUGCAC | -0.119868 |
| PAX3,7.p2 | -0.115442 |
| NANOG.p2 | 0.104343 |
| ATF5_CREB3.p2 | 0.094706 |
| GAUAUGU | -0.084700 |
| SPI1.p2 | -0.081698 |
| TLX1..3_NFIC{dimer}.p2 | 0.080810 |
| UGACCUA | 0.076086 |
| MYFfamily.p2 | 0.069070 |
| FOS_FOS{B,L1}_JUN{B,D}.p2 | 0.068486 |
| HMGA1,2.p2 | 0.055254 |
| CUX2.p2 | -0.036901 |
| RUNX1..3.p2 | -0.033572 |
| SP1.p2 | -0.023642 |
| ACCCGUA | 0.014366 |

**Table-S3. Motif activity changes derived from mouse EMT models (sorted by absolute z-scores).**

| **Motif** | **Z-Score** |
| --- | --- |
| HNF1A.p2 | -18.319605 |
| TFAP2{A,C}.p2 | 13.139745 |
| HNF4A_NR2F1,2.p2 | -10.316133 |
| SNAI1..3.p2 | -9.802821 |
| NFY{A,B,C}.p2 | -9.260140 |
| NFIL3.p2 | -9.153861 |
| PATZ1.p2 | 9.037959 |
| GTF2A1,2.p2 | 8.129773 |
| E2F1..5.p2 | -8.107501 |
| XBP1.p3 | 7.993492 |
| SOX{8,9,10}.p2 | 7.578368 |
| JUN.p2 | 7.391577 |
| ESRRA.p2 | -7.092741 |
| ZEB1.p2 | -6.970979 |
| ZFP161.p2 | -6.773266 |
| RUNX1..3.p2 | 6.677799 |
| GAGGUAG | 6.640954 |
| TFCP2.p2 | 6.502995 |
| RXR{A,B,G}.p2 | 6.232599 |
| HBP1_HMGB_SSRP1_UBTF.p2 | 6.211917 |
| ZBTB16.p2 | -6.189857 |
| GATA1..3.p2 | 6.074720 |
| GUGCAAA | 5.968291 |
| SMAD1..7,9.p2 | 5.715875 |
| HES1.p2 | -5.703150 |
| GUAAACA | 5.658502 |
| DBP.p2 | -5.514266 |
| FOXA2.p3 | -5.288262 |
| FOSL2.p2 | 5.275215 |
| EN1,2.p2 | -5.001922 |
| STAT2,4,6.p2 | 4.958305 |
| YY1.p2 | -4.954490 |
| AGCACCA | 4.928008 |
| SOX2.p2 | 4.919780 |
| PRDM1.p3 | 4.820454 |
| TLX2.p2 | 4.812390 |
| MYB.p2 | -4.762636 |
| ATF6.p2 | 4.710928 |
| bHLH_family.p2 | 4.694504 |
| ZNF238.p2 | 4.656929 |
| PAX2.p2 | -4.622154 |
| RFX1..5_RFXANK_RFXAP.p2 | -4.531748 |
| UAUUGCU | 4.389541 |
| MYFfamily.p2 | 4.378652 |
| KLF12.p2 | 4.358762 |
| ZNF423.p2 | 4.227038 |
| NR1H4.p2 | 4.225841 |
| LMO2.p2 | -4.188487 |
| NFATC1..3.p2 | 4.178978 |
| HOX{A6,A7,B6,B7}.p2 | -4.123296 |
| GLI1..3.p2 | 4.085050 |
| AGCUGCC | -4.051388 |
| ESR1.p2 | 3.971369 |
| MEF2{A,B,C,D}.p2 | 3.903434 |
| NKX2-2,8.p2 | 3.857453 |
| IRF1,2,7.p3 | 3.845818 |
| FOXL1.p2 | 3.817527 |
| NR5A1,2.p2 | 3.787010 |
| ACAGUAC | 3.741890 |
| NFE2L2.p2 | 3.715022 |
| CEBPA,B_DDIT3.p2 | 3.686488 |
| POU1F1.p2 | -3.601421 |
| EBF1.p2 | 3.576447 |
| PAX5.p2 | -3.448711 |
| GFI1.p2 | -3.441597 |
| CCCUGAG | 3.391263 |
| ZBTB6.p2 | -3.359916 |
| ZIC1..3.p2 | 3.355679 |
| AAGUGCU | -3.342459 |
| AGGUAGU | -3.290403 |
| AUGGCAC | 3.285942 |
| UUGUUCG | -3.273784 |
| CCAGUGU | 3.246929 |
| FEV.p2 | 3.164132 |
| UCACAUU | -3.154298 |
| AGUGCAA | -3.125763 |
| UUGGUCC | 3.098766 |
| EGR1..3.p2 | -3.096638 |
| AGCAGCA | 3.094618 |
| SPIB.p2 | 3.082550 |
| FOX{I1,J2}.p2 | -3.035529 |
| UGUGCUU | 2.945159 |
| TFDP1.p2 | 2.916955 |
| RORA.p2 | -2.858638 |
| GGCAGUG | 2.851382 |
| TLX1..3_NFIC{dimer}.p2 | -2.774303 |
| NANOG{mouse}.p2 | -2.761615 |
| TFAP2B.p2 | 2.759048 |
| BPTF.p2 | -2.699265 |
| EP300.p2 | 2.671825 |
| EOMES.p2 | -2.609712 |
| CTCF.p2 | 2.570247 |
| NKX3-2.p2 | -2.518638 |
| IKZF1.p2 | -2.497992 |
| ARID5B.p2 | 2.487411 |
| CREB1.p2 | -2.472282 |
| AUUGCAC | -2.444454 |
| REST.p3 | 2.442849 |
| GGAAUGU | 2.441918 |
| MTF1.p2 | 2.431261 |
| SOX5.p2 | -2.409517 |
| FOX{D1,D2}.p2 | 2.387016 |
| AAGGUGC | 2.379286 |
| NKX2-3_NKX2-5.p2 | 2.367255 |
| ALX1.p2 | -2.352391 |
| UGCAUAG | 2.323399 |
| ZNF148.p2 | -2.252424 |
| UCACAGU | -2.232014 |
| HIC1.p2 | 2.222399 |
| PBX1.p2 | -2.212457 |
| ATF5_CREB3.p2 | -2.152149 |
| KLF4.p3 | -2.127946 |
| ACAGUAU | -2.127841 |
| EHF.p2 | -2.110093 |
| IKZF2.p2 | 2.100513 |
| GAUUGUC | -2.083073 |
| DMAP1_NCOR{1,2}_SMARC.p2 | -2.044785 |
| SPZ1.p2 | 2.036622 |
| MYOD1.p2 | -2.020948 |
| AAUACUG | 2.010256 |
| FOX{F1,F2,J1}.p2 | 1.951040 |
| STAT5{A,B}.p2 | 1.944012 |
| UGAAAUG | -1.941875 |
| ELK1,4_GABP{A,B1}.p3 | -1.931232 |
| LHX3,4.p2 | 1.906492 |
| SRY.p2 | 1.894688 |
| ZNF384.p2 | -1.824031 |
| AGUGGUU | 1.818739 |
| MYBL2.p2 | 1.784815 |
| GATA6.p2 | -1.762058 |
| TFAP4.p2 | 1.760603 |
| TAL1_TCF{3,4,12}.p2 | 1.682352 |
| AGCUUAU | -1.675293 |
| ONECUT1,2.p2 | -1.633590 |
| UCAAGUA | 1.596472 |
| FOX{C1,C2}.p2 | -1.549272 |
| FOXD3.p2 | 1.515649 |
| UUGGCAA | -1.504195 |
| HLF.p2 | 1.501009 |
| HMX1.p2 | -1.489057 |
| ADNP_IRX_SIX_ZHX.p2 | -1.482936 |
| UCCAGUU | -1.457275 |
| SRF.p3 | 1.452882 |
| PRRX1,2.p2 | 1.451130 |
| NANOG.p2 | 1.449412 |
| NKX6-1,2.p2 | 1.447207 |
| NFIX.p2 | -1.431007 |
| AHR_ARNT_ARNT2.p2 | -1.397611 |
| ZNF143.p2 | -1.383203 |
| ACUGGCC | -1.362517 |
| HSF1,2.p2 | -1.360968 |
| UCCCUUU | -1.357800 |
| TBX4,5.p2 | -1.348500 |
| NFE2L1.p2 | 1.343626 |
| GCAGCAU | 1.331934 |
| CAGUGCA | 1.306031 |
| STAT1,3.p3 | 1.298060 |
| AACAGUC | 1.292869 |
| HAND1,2.p2 | 1.287578 |
| UUUUUGC | -1.280302 |
| GFI1B.p2 | -1.267603 |
| FOXQ1.p2 | -1.246515 |
| GZF1.p2 | 1.202089 |
| ELF1,2,4.p2 | 1.198595 |
| NRF1.p2 | -1.197642 |
| T.p2 | 1.190501 |
| GGACGGA | 1.116742 |
| CUX2.p2 | -1.071701 |
| HOXA9_MEIS1.p2 | -1.064329 |
| LEF1_TCF7_TCF7L1,2.p2 | 1.056026 |
| TGIF1.p2 | -1.034514 |
| UAAUGCU | -0.996918 |
| HOX{A4,D4}.p2 | -0.983296 |
| POU5F1.p2 | -0.980397 |
| PPARG.p2 | -0.965255 |
| AIRE.p2 | 0.924218 |
| GUAACAG | 0.910750 |
| NFKB1_REL_RELA.p2 | -0.905472 |
| NR6A1.p2 | 0.882506 |
| CDC5L.p2 | -0.862852 |
| TEAD1.p2 | 0.852762 |
| GGAGUGU | -0.843095 |
| FOXP3.p2 | -0.838785 |
| AUGACAC | -0.812233 |
| GCUGGUG | 0.807405 |
| CUCCCAA | 0.798569 |
| RXRA_VDR{dimer}.p2 | 0.780997 |
| AR.p2 | 0.736254 |
| BACH2.p2 | -0.736097 |
| AACCUGG | -0.734849 |
| TP53.p2 | -0.723473 |
| UAAGACU | -0.715628 |
| SPI1.p2 | 0.704271 |
| GUAGUGU | -0.702330 |
| RREB1.p2 | -0.698246 |
| HOX{A5,B5}.p2 | 0.693836 |
| GTF2I.p2 | 0.678249 |
| MSX1,2.p2 | 0.626156 |
| POU5F1_SOX2{dimer}.p2 | 0.624742 |
| AAUCUCU | -0.614284 |
| UGCAUUG | -0.601594 |
| ATF2.p2 | -0.587577 |
| MAZ.p2 | -0.569641 |
| GCUACAU | -0.562374 |
| UAAGACG | -0.562314 |
| AGCAGCG | 0.559258 |
| AUGGCUU | 0.542322 |
| CGUGUCU | 0.538620 |
| GUCAGUU | 0.505634 |
| FOS_FOS{B,L1}_JUN{B,D}.p2 | 0.498148 |
| CRX.p2 | -0.489037 |
| ACUGCAU | -0.478429 |
| POU6F1.p2 | -0.463786 |
| CCAGCAU | 0.459199 |
| TCF4_dimer.p2 | 0.445521 |
| MAFB.p2 | 0.424878 |
| AACACUG | -0.405680 |
| TFEB.p2 | -0.371070 |
| CDX1,2,4.p2 | -0.369270 |
| TBP.p2 | 0.349531 |
| CGUACCG | -0.336247 |
| SP1.p2 | 0.333897 |
| GAGAUGA | 0.331195 |
| PAX6.p2 | -0.323456 |
| EWSR1-FLI1.p2 | -0.319026 |
| GGCUCAG | 0.318037 |
| AACGGAA | 0.309046 |
| POU2F1..3.p2 | 0.299161 |
| AAUCUCA | -0.293698 |
| GAUCAGA | 0.292939 |
| CACAGUG | 0.279905 |
| PAX8.p2 | -0.271819 |
| ATF4.p2 | -0.259694 |
| GAUAUGU | 0.255698 |
| AAGGCAC | -0.242607 |
| HIF1A.p2 | -0.238108 |
| PAX4.p2 | 0.236628 |
| CUUUGGU | -0.231595 |
| NKX2-1,4.p2 | -0.226075 |
| NKX3-1.p2 | 0.225196 |
| UGACCUA | -0.218150 |
| PDX1.p2 | 0.209241 |
| GGCAAGA | -0.203007 |
| PAX3,7.p2 | -0.200453 |
| POU3F1..4.p2 | 0.190423 |
| MZF1.p2 | 0.186999 |
| FOXO1,3,4.p2 | -0.166946 |
| GGAAGAC | 0.161723 |
| AACCGUU | -0.161281 |
| GAGAACU | -0.154159 |
| PITX1..3.p2 | -0.139404 |
| VSX1,2.p2 | -0.132444 |
| SOX17.p2 | -0.122967 |
| UGUGCGU | 0.122439 |
| NFE2.p2 | 0.119513 |
| ARNT_ARNT2_BHLHB2_MAX_MYC_USF1.p2 | -0.105121 |
| ACCCUGU | -0.094109 |
| UUGGCAC | 0.088067 |
| EVI1.p2 | -0.087064 |
| CUACAGU | 0.079026 |
| RXRG_dimer.p3 | -0.061463 |
| RBPJ.p2 | -0.057384 |
| AAAGUGC | -0.054632 |
| ACCCGUA | -0.043866 |
| ACAUUCA | 0.043493 |
| NHLH1,2.p2 | -0.033172 |
| HMGA1,2.p2 | -0.032751 |
| ETS1,2.p2 | 0.031012 |
| AAUGCCC | -0.025423 |
| CCUUCAU | 0.019178 |
| CAGCAGG | 0.016615 |
| SREBF1,2.p2 | 0.010177 |
| FOXN1.p2 | -0.009457 |
| NR3C1.p2 | -0.006974 |

**Table-S4. Fisher test of EMT signature genes represented in differentially expressed genes in response to TFAP2A overexpression.**

| EMT Signature Set[^5^](#_ENREF_5)/Tfap2a | EMT Sign. Genes:  Changing | EMT Sign. Genes: Not  Changing | All Genes:  Changing | All Genes:  Not Changing | Pval |
| --- | --- | --- | --- | --- | --- |
| Up/Up | 18 | 42 | 2007 | 12798 | 0.0007559 |
| Up/Down | 6 | 55 | 1538 | 13267 | 0.6181231 |
| Down/Down | 19 | 87 | 1538 | 13267 | 0.0130989 |
| Down/Up | 27 | 79 | 2007 | 12798 | 0.0008046 |

**Figure-S1: Expression Profile of AP-2 family members.** mRNAseq libraries from the following conditions NMuMG CTRL cells or cells treated for 14 days with growth factor, were generated and read coverage of the genomic region spanning the genes from the family of AP-2 transcription factors is shown in a mouse genome browser (www. clipz.unibas.ch [^8^](#_ENREF_8)). High densities of the reads (reads per million unit) are only present for the Tfap2a gene, while sporadic reads can be assigned to the other family members suggesting they have no or little expression. Mapping, annotation and visualization of deep-sequencing data was done with the CLIPZ server ^[8](#_ENREF_8)^.

pCLX-GFP

pCLX-TFAP2A

GAPDH

TFAP2A

**Figure-S2: Overexpression of TFAP2A protein is detected by Western Blot.**

Lysates from NMuMG cells stably transduced with pCLX-TFAP2A or pCLX-GFP treated for 72 hours with 2 μg/mL doxycycline (Dox) or not (Nox) were probed for TFAP2A expression by WB. GAPDH is used as normalization control. Overexpression of TFAP2A is detectable only in the pCLX-TFAP2A doxycycline- induced cells.

cc

a

b

**
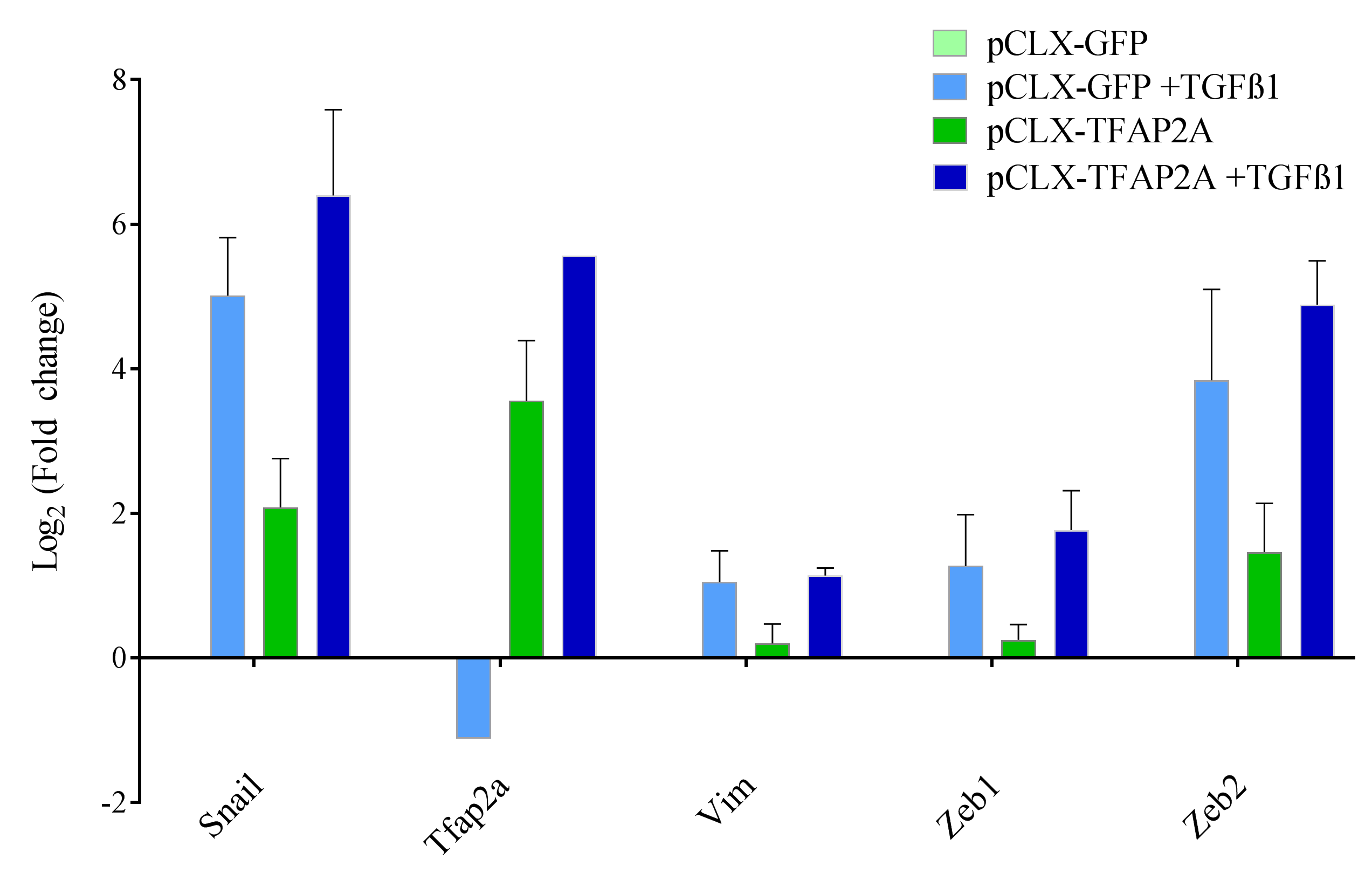
**

**Figure-S3: TFAP2A overexpression in NMuMG modulates epithelial plasticity.**

Quantitative RT-PCR of EMT markers on extracts from the doxycycline induced pCLX-TFAP2A or pCLX-GFP cell lines, treated or not with 2 ng/mL of TGFβ1 for 72 hours. The results represented in the figure are the mean values from three experiments for cells non-treated with growth factor (light and dark green) and two independent experiments in the case of TGFβ1-induced samples. a) EMT markers that do not show differential expression upon TFAP2A induction. b) Set of genes (Stg3gal5, Tln1, Tnc, Zyx) involved in focal adhesion that are unregulated in both TGFβ1-induced EMT and upon TFAP2A induction. In addition, the St3gal5 gene that was recently involved in cell adhesion downstream of TGFβ1 signaling and Zeb1 is also significantly changing in both conditions ^[9](#_ENREF_9)^. The genes that displayed significant enrichment in the TFAP2A cell line versus the GFP cell line, as estimated from the performed one-tail paired *t*-test, are indicated with asterix (*) for p<0.05 and (**) for p < 0.01, respectively. (c) Quantitative RT-PCR of EMT markers (TFs) in extracts from the doxycycline-induced pCLX-GFP and pCLX-TFAP2A cell lines, treated or not with 2 ng/mL of TGFβ1 for 72 hours. Shown are the means from three experiments on cells not treated with growth factor (light and dark green) and two independent experiments in TGFβ1-induced cells. TFAP2A expression in TGFβ1-induced cells was only measured once. TFAP2A overexpression is apparent in both TFAP2A-induced samples (dark green and dark blue) but is not induced in cell treated with TGFβ1 alone (light blue). The EMT-inducing TFs have increased expression upon TFAP2A induction.

**Figure-S4: Correlation between TFAP2A/C activity and mRNA expression levels during EMT time course.** mRNA-seq samples in two replicates form independent wells were prepared from a time course of NMuMG cells treated for 14 days with 2 ng/mL of TGFβ1, and the data was consequently analyzed with MARA [^10^](#_ENREF_10). The figure emphasizes the correlation between TFAP2A/C transcriptional activity and mRNA expression levels during the time course.

**
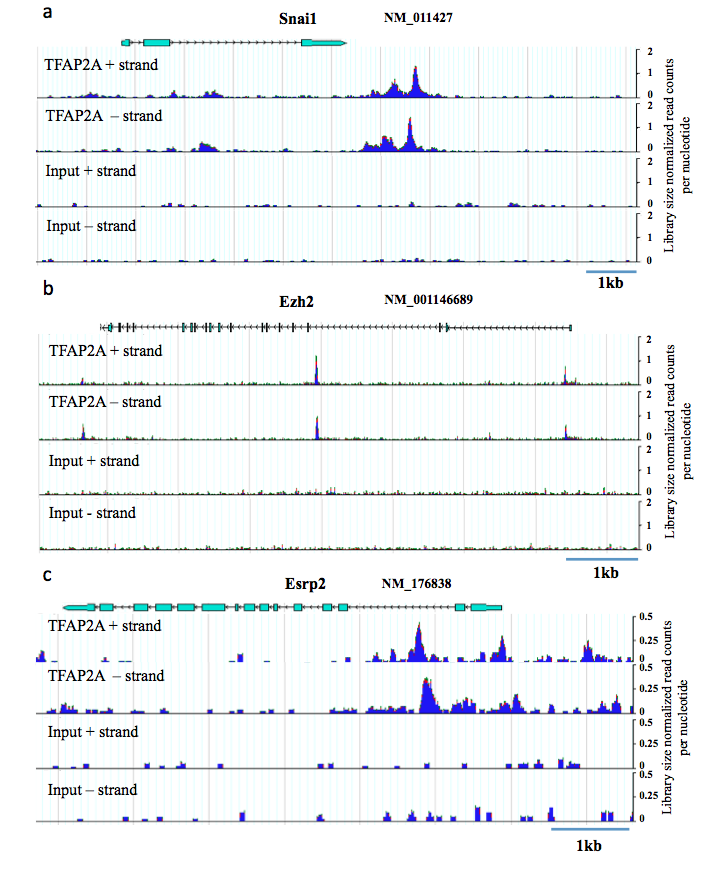
**

a

**2**

**1**

b

c

**
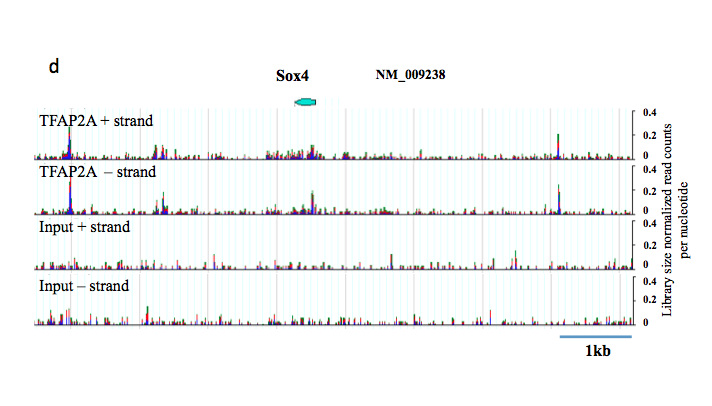
**

d

**Figure-S5: TFAP2A binds directly to crucial regulars of**

**EMT.** ChIP-seq libraries from TFAP2A ChIP or Input were generated and the coverage of the genomic region spanning Snai1 (a), Ezh2 (b), Esrp2 (c), Sox4 (d) genes by reads is shown in a mouse genome browser ([www.clipz.unibas.ch](http://www.clipz.unibas.ch) and [^8^](#_ENREF_8)). The results of a representative experiment are presented. Mapping, annotation and visualization of deep-sequencing data was done with the ClipZ server ^[8](#_ENREF_8)^.

**
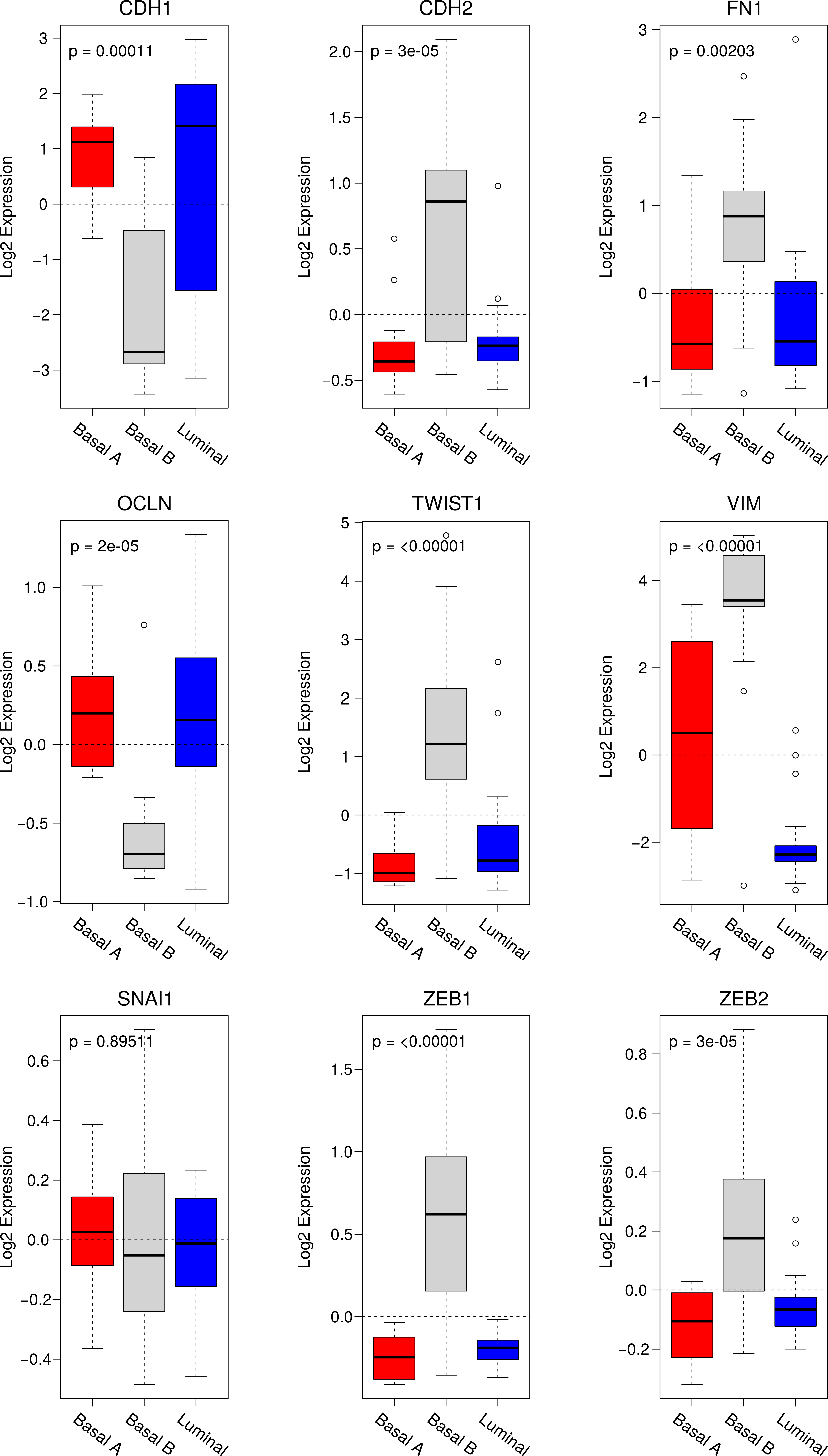
**

**Figure-S6: EMT marker expression in breast cancers.**

Box plots of marker gene expression in a panel of breast cancer cell lines grouped in the basal A (red), basal B (grey) and luminal (blue) subgroups based on the annotation from Neve et al. [^11^](#_ENREF_11) All plots were generated with the GOBO online tool [^12^](#_ENREF_12).


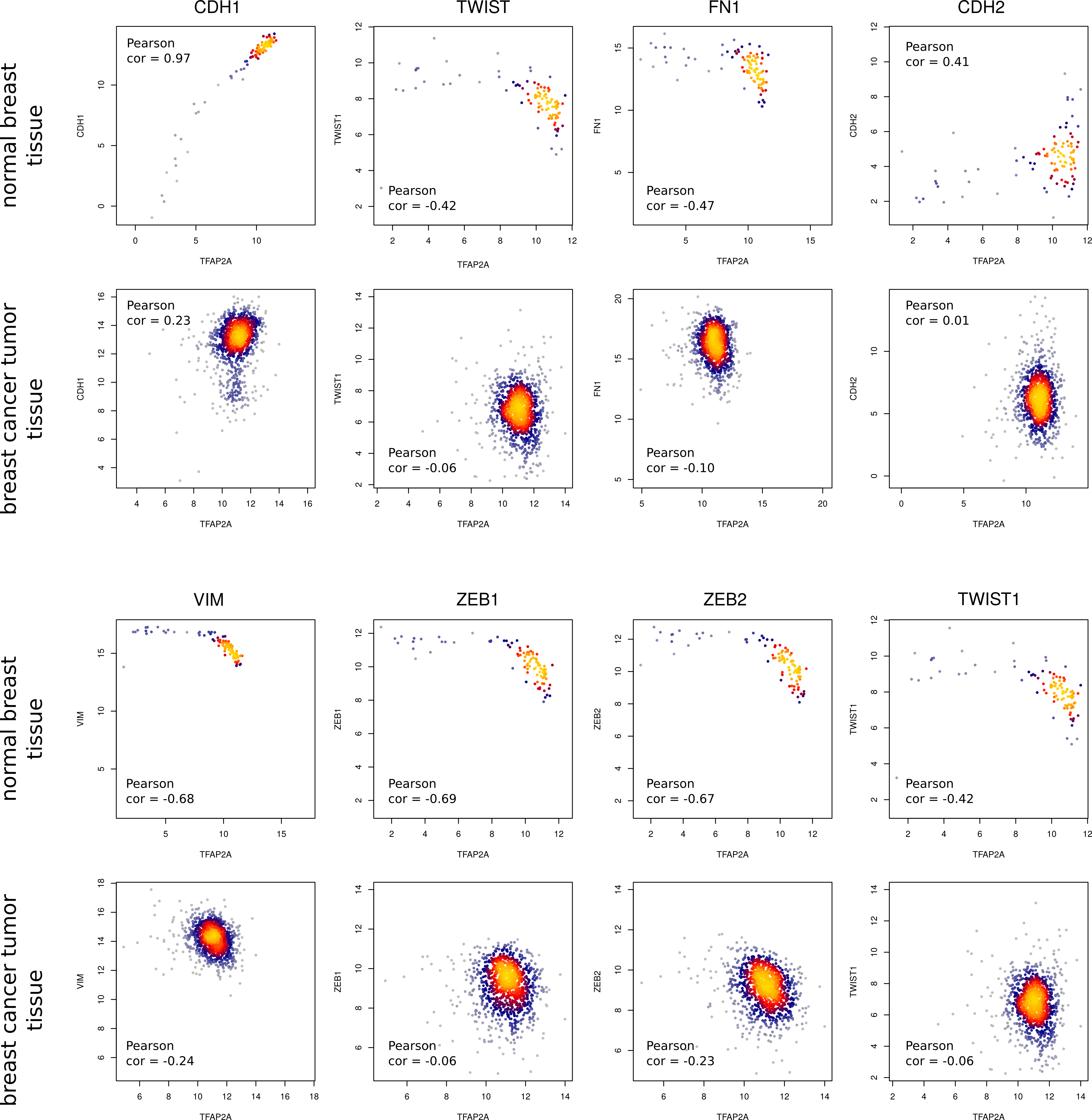


**Figure-S7: Correlation of log2 expression values of TFAP2A and EMT marker genes in normal and tumor breast tissues.**

The plots show the correlation of TFAP2A expression with the expression of the indicated epithelial and mesenchymal markers in normal breast tissue samples (n = 98) and of breast cancer samples (n = 1080). Normalized expression values of the indicated genes were obtained from The Cancer Genome Atlas project in Breast Invasive Carcinoma (TCGA-BRCA), more specifically from the Broad Institute TCGA Genome Data Analysis Center (2016): Analysis-ready standardized TCGA data from Broad GDAC Firehose 2016_01_28 run. Broad Institute of MIT and Harvard. Dataset. https://doi.org/10.7908/C11G0KM9).

**Supplementary methods**

**Analysis of mRNA-Sequencing data**

Mapping and annotation of sequencing reads was done using the CLIPZ webserver ^[8](#_ENREF_8)^. Differential gene expression analysis was conducted using the Bioconductor DESeq package [^13^](#_ENREF_13), whereat genes with an adjusted p-value < 0.05 were considered differentially expressed. Gene ontology (GO) analysis of differentially expressed genes was done with the topGO package [^14^](#_ENREF_14) using the ”weight01” algorithm with a node size of one and ”Fisher” statistics.

**Analysis of EMT signature genes enrichment**

The differentially expressed genes between NMuMG cells that overexpress TFAP2A and NMuMG cells that overexpress GFP were compared to the EMT signature set of up- or downregulated genes [^5^](#_ENREF_5). Those with an adjusted p-value < 0.05 and following the defined direction of modulation (Up or Down) were considered as changing, whereas those with an adjusted p-value > 0.05 or not following the defined trend were defined as non-changing. In this manner four categories of genes sets comparing the signature gene set vs the genes differentially expressed upon TFAP2A overexpression were created Up/Up, Up/Down, Down/Down and Down/Up and for each category the number of genes Changing and Not-Changing is calculated. A Fisher statistics was used to calculate the enrichment of the genes in each of the four categories as compared to all genes up- or downregulated upon TFAP2A overexpression (<http://www.quantitativeskills.com/sisa/statistics/fisher.htm>), the one sided p-value p(O>=E) is represented.

**REFERENCES**

1 Kreitzer, F. R. *et al.* A robust method to derive functional neural crest cells from human pluripotent stem cells. *Am J Stem Cells* **2**, 119-131 (2013).

2 Feuerborn, A. *et al.* The Forkhead factor FoxQ1 influences epithelial differentiation. *J Cell Physiol* **226**, 710-719, doi:10.1002/jcp.22385 (2011).

3 Tiwari, N. *et al.* Klf4 is a transcriptional regulator of genes critical for EMT, including Jnk1 (Mapk8). *PLoS One* **8**, e57329, doi:10.1371/journal.pone.0057329 (2013).

4 Evseenko, D. *et al.* Mapping the first stages of mesoderm commitment during differentiation of human embryonic stem cells. *Proc Natl Acad Sci U S A* **107**, 13742-13747, doi:10.1073/pnas.1002077107 (2010).

5 Taube, J. H. *et al.* Core epithelial-to-mesenchymal transition interactome gene-expression signature is associated with claudin-low and metaplastic breast cancer subtypes. *Proc Natl Acad Sci U S A* **107**, 15449-15454, doi:10.1073/pnas.1004900107 (2010).

6 Diepenbruck, M. *et al.* Tead2 expression levels control the subcellular distribution of Yap and Taz, zyxin expression and epithelial-mesenchymal transition. *J Cell Sci* **127**, 1523-1536, doi:10.1242/jcs.139865 (2014).

7 Brunskill, E. W. *et al.* A gene expression atlas of early craniofacial development. *Dev Biol* **391**, 133-146, doi:10.1016/j.ydbio.2014.04.016 (2014).

8 Khorshid, M., Rodak, C. & Zavolan, M. CLIPZ: a database and analysis environment for experimentally determined binding sites of RNA-binding proteins. *Nucleic Acids Res* **39**, D245-252, doi:10.1093/nar/gkq940 (2011).

9 Mathow, D. *et al.* Zeb1 affects epithelial cell adhesion by diverting glycosphingolipid metabolism. *EMBO Rep* **16**, 321-331, doi:10.15252/embr.201439333 (2015).

10 Balwierz, P. J. *et al.* ISMARA: automated modeling of genomic signals as a democracy of regulatory motifs. *Genome Res* **24**, 869-884, doi:10.1101/gr.169508.113 (2014).

11 Neve, R. M. *et al.* A collection of breast cancer cell lines for the study of functionally distinct cancer subtypes. *Cancer Cell* **10**, 515-527, doi:10.1016/j.ccr.2006.10.008 (2006).

12 Ringner, M., Fredlund, E., Hakkinen, J., Borg, A. & Staaf, J. GOBO: gene expression-based outcome for breast cancer online. *PLoS One* **6**, e17911, doi:10.1371/journal.pone.0017911 (2011).

13 Anders, S. & Huber, W. Differential expression analysis for sequence count data. *Genome Biol* **11**, R106, doi:10.1186/gb-2010-11-10-r106 (2010).

14 topGO: Enrichment analysis for Gene Ontology. (2010).
